# Adaptive Gene Regulation in the Striatum of RGS9-Deficient Mice

**DOI:** 10.1371/journal.pone.0092605

**Published:** 2014-03-24

**Authors:** Kathy Busse, Rainer Strotmann, Karl Strecker, Florian Wegner, Vasudharani Devanathan, Antje Gohla, Torsten Schöneberg, Johannes Schwarz

**Affiliations:** 1 Department of Neurology, Medical Faculty, University of Leipzig, Leipzig, Germany; 2 Institute of Biochemistry, Medical Faculty, University of Leipzig, Leipzig, Germany; 3 Translational Centre of Regenerative Medicine (TRM), University of Leipzig, Leipzig, Germany; 4 Institute of Pharmacology and Toxicology, Eberhard Karls University of Tübingen, Tübinge, Germany; 5 Rudolf Virchow Centre of Experimental Biomedicine, Julius Maximilians University of Würzburg, Würzburg, Germany; 6 Department of Neurology, Technical University of Munich, Munich, Germany; Indiana University School of Medicine, United States of America

## Abstract

**Background:**

RGS9-deficient mice show drug-induced dyskinesia but normal locomotor activity under unchallenged conditions.

**Results:**

Genes related to Ca^2+^ signaling and their functions were regulated in RGS9-deficient mice.

**Conclusion:**

Changes in Ca^2+^ signaling that compensate for RGS9 loss-of-function can explain the normal locomotor activity in RGS9-deficient mice under unchallenged conditions.

**Significance:**

Identified signaling components may represent novel targets in antidyskinetic therapy. The long splice variant of the regulator of G-protein signaling 9 (RGS9-2) is enriched in striatal medium spiny neurons and dampens dopamine D2 receptor signaling. Lack of RGS9-2 can promote while its overexpression prevents drug-induced dyskinesia. Other animal models of drug-induced dyskinesia rather pointed towards overactivity of dopamine receptor-mediated signaling. To evaluate changes in signaling pathways mRNA expression levels were determined and compared in wild-type and RGS9-deficient mice. Unexpectedly, expression levels of dopamine receptors were unchanged in RGS9-deficient mice, while several genes related to Ca^2+^ signaling and long-term depression were differentially expressed when compared to wild type animals. Detailed investigations at the protein level revealed hyperphosphorylation of DARPP32 at Thr34 and of ERK1/2 in striata of RGS9-deficient mice. Whole cell patch clamp recordings showed that spontaneous synaptic events are increased (frequency and size) in RGS9-deficient mice while long-term depression is reduced in acute brain slices. These changes are compatible with a Ca^2+^-induced potentiation of dopamine receptor signaling which may contribute to the drug-induced dyskinesia in RGS9-deficient mice.

## Introduction

Drug-induced dyskinesia is an important clinical challenge in both Parkinsonian patients treated with L-DOPA and/or dopamine agonists and patients receiving neuroleptics. Since both classes of drugs primarily act via dopamine receptors, it is generally accepted that modulation of downstream signaling of these molecules forms the primary event in the pathophysiology of such movement disorders [1]. However, animal models for drug-induced dyskinesia to dissect involved signaling pathways downstream of the dopamine receptors are sparse. Cenci and colleagues characterized l-DOPA-induced dyskinesia (LID) in rats that were first rendered hemiparkinsonian via unilateral midbrain injections of 6-hydroxydopamine and subsequently treated with rather high doses of l-DOPA [2]. Dopamine receptors are pharmacologically differentiated into the dopamine D1 (D1R) and the dopamine D2 receptor (D2R) families [3]. While the former activates adenylyl cyclases (AC) via Gα_s_ and Gα_olf_ the latter inhibits AC acting via Gα_i_ (AC types I, V, VI) and Gα_o_ (AC type I) [4]–[5]. D2R agonists can provoke dyskinesia in clinical stages of dopamine deficiency [1], [6]–[7] and inhibition of D1R signaling prevented dyskinesia in hemiparkinsonian rats [8]. Further evidence that excessive dopamine receptor signaling is involved in dyskinesia was provided by rodent and primate studies overexpressing GRK6 [9]. GRK6 is a G protein-coupled receptor kinase which controls desensitization of dopamine receptors.

RGS9-deficient mice represent a genetic animal model for the phenotype of drug-induced dyskinesia [10]–[12]. Lack of this accessory protein of GPCR signaling seems to predispose for l-DOPA and neuroleptic-induced dyskinesias [10]. Regulators of G-protein signaling (RGS) form a heterogeneous family of GTPase activating proteins (GAP) that in addition to accelerating G-protein turnover have multiple other functions [13]. RGS proteins regulate a variety of Gα subunits, but do not interact with stimulatory Gα_s_ proteins. However, Gβ5 is an obligate binding partner to RGS9-2 and this dimeric complex has been recently shown to directly modulate adenylyl cyclase function [14]. The striatum is the major target of dopaminergic pathways in the CNS and contains most of the postsynaptic dopamine receptors. It also bears the highest expression levels of the long splice variant of the ninth member of the RGS family (RGS9-2) [15]–[16]. Modulation of striatal dopaminergic transmission by RGS9-2 is most likely restricted to D2R signaling as D1R are mainly coupled to Gα_olf_ in striatum but also Gα_s_ in other tissues [16]–[17].

Mice lacking RGS9 display increased abnormal involuntary movements following dopamine depletion and subsequent administration of dopamine receptor agonists or L-DOPA [10]. Consistently, overexpression of RGS9 in dyskinetic non-human primates resulted in a reduction of such L-DOPA-induced dyskinesia [12]. The functional interaction of D2R and RGS9-2 is supported by the increased fraction of high affinity D2R present in RGS9-deficient mice [18]. Accordingly, in a rat model of schizophrenia with sensitization to amphetamine and in patients suffering from schizophrenia, reduced levels of RGS9 were detected [11]. It is therefore very likely that RGS9-2 has important functional effects on D2R-mediated signaling of striatal medium spiny neurons (sMSN) most likely mediated by accelerating the GTPase activity of G proteins.

Although an enhanced striatal D2R-dependent dopaminergic signal transduction and some deficits in working memory and motor coordination [16], RGS9-deficient mice show an almost normal motor phenotype under unchallenged conditions [19]. However, in RGS9-deficient mice that are treated with the D2R-specific agonist quinpirole following pretreatment with reserpine exhibit pronounced dyskinesia that is absent in wild-type (wt) mice [10]. Under the circumstance of reserpine-dependent depletion of dopaminergic transmission, striatal dysfunction becomes unmasked that is normally balanced by compensatory changes on the functional or gene regulation level. A similar situation may be present, when drug-induced dyskinesia develops either in patients with Parkinson’s disease or schizophrenia receiving long-term therapy with L-DOPA or neuroleptics. The analysis of compensatory gene regulation in functional dopaminergic dysbalance may thus help both understanding the pathophysiology of drug-induced dyskinesia and identifying novel targets for antidyskinetic therapy.

Therefore, we screened for compensatory changes in striata of RGS9-deficient mice. Based on the function of RGS9 in D2R signaling we hypothesized that RGS9-deficient mice should display decreased cAMP signaling as a consequence of overactive D2R. We found no evidence for decreased cAMP signaling but rather detected molecular changes in Ca^2+^ signaling. Our data point towards a Ca^2+^-induced potentiation of dopamine receptor signaling that may contribute to drug-induced dyskinesia in RGS9-deficient mice.

## Materials and Methods

### RGS9-deficient Mouse Strain and Mouse Genotyping

The generation and initial characterization of the RGS9-deficient mice was reported previously [20]. All animal experiments were conducted with mice on a C57Bl6 background (>12 generations) and in accord with accepted standards of animal care (NIH guidelines) and approved by the respective regional government agency of Saxony (Regierungspräsidium Leipzig, TV 42/08).

Genotyping was done by PCR using mouse tail DNA and three primers. The two reverse primers complementary annealed either to MC1neopA-cassette in the inactivated RGS9 gene (5′- GGCTATGACTGGGCACAACA -3′) or to the sequence that is substituted by MC1neopA-cassette in RGS9-deficient mice (5′- ACAGCGGAAGCCATAGAGGA -3′). The attribution of the genotype resulted from the different size of the PCR product with the forward-primer (5′- TTGGGCTCTTGCTCGTGTTA -3′) (94°C for 2 min; 35 cycles of 94°C for 30 sec, 61°C for 30 sec, 72°C for 90 sec; 72°C for 10 min).

### RNA Isolation and Microarray Expression Analysis

Striata (dorsal and ventral part) of 3-month-old wt and RGS9-deficient male mice were isolated by microdissection and total RNA was isolated using TRIzol reagent (Life Technologies, Carlsbad, USA) according to the manufacturer’s instructions. The RNA was further purified using the SV Total RNA Isolation System (Promega, Mannheim, Germany) according to the manual. For microarray analysis, RNA integrity and concentration were quantified on an Agilent 2100 Bioanalyzer (Agilent Technologies, Palo Alto, USA) using the RNA 6.000 LabChip Kit (Agilent Technologies, Palo Alto, USA).

For reverse transcription (SuperScript II, Life Technologies, Carlsbad, USA) of total striatal RNA (1 μg) from 5 wt and 5 RGS9-deficient mice, an oligo-dT primer containing a T7 RNA polymerase promoter site (Genset SA, Paris, France) was used. After purification of cDNA by phenol-chloroform extraction, *in vitro* transcription using ENZO BioArray RNA transcript labeling kit (Affymetrix, Santa Clara, USA) was performed. Unincorporated nucleotides were removed using the RNeasy kit. Fragmented cRNA was hybridized to GeneChip Mouse Genome Arrays 430 2.0 (Affymetrix, Santa Clara, USA) at the IZKF Leipzig microarray core facility.

The expression data were normalized with the Microarray Suite 5 (MAS5) algorithm using the R software package (http://www.r-project.org/). The annotation of the probe sets was obtained from the Affymetrix homepage. The datasets were filtered for transcripts that were detected as present in at least three microarrays of one group (wt or RGS9-deficient mice) [21]. Statistical testing was done using a two-tailed Student’s t-test. For gene ontology profiling, MAS5 normalized data with P≤0.01 were subjected to the Onto Express algorithm [22]–[23]. The settings were: hypergeometric distribution, correction for false discovery rate (fdr). The MAS5 processed data were also analyzed using the Gene Set Enrichment Analysis (GSEA) algorithm [24]. Gene sets were obtained from the Kyoto Encyclopedia of Genes and Genomes database (KEGG) [25]. GSEA settings were: 500 phenotype permutations, datasets collapsed to gene symbols and weighted enrichment with signal-to-noise metric.

### Preparation of cDNA and Quantification by Real-time PCR

For quantitative real-time PCR analysis (qPCR), Platinum SYBR Green qPCR Supermix (Life Technologies, Carlsbad, USA), cDNA from 30 ng total RNA, 0.6 μM forward and reverse primers and 100 nM ROX (5-carboxy-X-rhodamine, passive references dye) were used. Oligonucleotide primers (Table S1 in File S1) were designed using the Primer3 software [26] to flank intron sequences if possible. PCR was performed in an MX 3000P instrument (Stratagene, La Jolla, USA) using the following protocol: 2 min 50°C, 2 min 95°C and 50 cycles of 15 s 95°C, 30 s 60°C. To confirm the presence of a single amplicon, product melting curves were recorded. The correct size of the amplicons was asserted by agarose gel electrophoresis and the identity verified by restriction enzyme cleavage or sequencing for at least 10% of the amplicons. Threshold cycle (C_T_) values were set within the exponential phase of the PCR. Data were normalized to β2-microglobulin and ΔC_T_ values were used to calculate the relative expression levels [27]. Gene regulation was statistically evaluated by subjecting the ΔΔC_T_ values (ΔC_T_ RGS9-deficient minus ΔC_T_ wt) to a two-tailed Student’s t-test assuming equal variances. Gene regulation values are given as 2^−ΔΔC^_T_ ± SEM.

### Western Blot Analysis

Striata of wt and RGS9-deficient mice were homogenized by sonication in phosphate buffered saline (PBS) containing 1 mM Na_2_EDTA and a protease inhibitor cocktail (Roche, Basel, Switzerland). The protein content was determined using Bradford reagent (Bio-Rad, Hercules, USA). 50 μg or 20 μg total protein were subjected to 10 or 12.5% SDS-PAGE and transferred onto nitrocellulose membrane (Hybond-C Extra, Amersham, Piscataway, USA). After blocking with 5% non-fat dry milk in Tris-buffered saline (50 mM Tris, pH 7.6, 150 mM NaCl) containing 0.1% Tween-20 (TBST) for 2 h at 4°C, the blots were incubated with primary antibody for 1 h at room temperature or over night at 4°C. The primary antibodies were: anti-RGS9 (Santa Cruz Biotechnology Inc., Dallas, USA), anti-phosphoDARPP32 (Thr34) and (Thr75) (Cell Signaling, Boston, USA), anti-DARPP32 (Cell Signaling, Boston, USA), anti-phospho-p44/42 MAPK (Cell Signaling, Boston, USA), anti-MAPK (Zymed Laboratories Inc., San Francisco, USA), anti-CaMKIIβ (ProteinTech Group Inc., Chicago, USA), anti-phospho-Thr286 CaMKII (PhosphoSolutions, Aurora, USA), anti-CaMKIIγ (Santa Cruz Biotechnology Inc., Dallas, USA), anti-GluR2 (Santa Cruz Biotechnology Inc., Dallas, USA) and anti-actin (Molecular Probes, Eugene, USA). The dilution of primary antibodies was 1∶1000, except for anti-CaMKIIγ (1∶500) and anti-GluR2 (1∶200). After extensive washing in TBST, membranes were incubated with horseradish peroxidase-linked anti-mouse, -goat or -rabbit secondary antibody (Dianova, Hamburg, Germany) diluted 1∶10,000 in TBST for 1 h at room temperature. After extensive washing with TBST, immunostaining was performed using ECL Western Blotting Substrate (PIERCE, Rockford, USA). Band densitometry was carried out using the Scion Image software (Scion, Frederick, USA). Values are given as mean ± SEM. Protein concentrations were statistically evaluated using a two-tailed Student’s t-test assuming equal variances.

### Cyclic AMP Measurements in Acute Striatal Samples

Striatal samples of defined size were dissected and individually transferred into ice-cold stimulation buffer (0.1% BSA, 0.5 mM IBMX, 5 mM HEPES in HBSS, pH 7.4) containing forskolin at the given concentrations. To investigate the influence of D2R signaling on adenylyl cyclase activity, 10 μM forskolin and quinpirole between 0.01 and 100 μM were added. After stimulation at 37°C for 1 h, samples were transferred into 500 μl ice-cold 0.1 N HCl, homogenized by sonication for 15 sec at 4°C (1 cycle, 100% amplitude, UP-50 H, Hielscher Ultrasonics) and incubated on ice for 30 min. Homogenates were centrifuged for 10 min and 14000×g at 4°C. Supernatants were transferred into new reaction tubes and dried at 56°C over night. Pellets were dissolved in 100 μl lysis buffer (5 mM HEPES, pH 7.4, 0.1% BSA, 1 mM IBMX, 0.3% Tween-20) and the cAMP concentration was measured in an AlphaScreen-based assay (Perkin Elmer, Waltham, USA) according to the manufacturer’s instructions.

### Electrophysiological Recordings

Coronal slices (300 μm thick) containing the striatum were prepared from the brains of RGS9-deficient mice and their wt littermates. Slices were superfused with an artificial cerebrospinal fluid (ACSF) containing (in mM): 125 NaCl, 2.5 KCl, 2 CaCl_2_, 1 MgCl_2_, 26 NaHCO_3_, 1.25 NaH_2_PO_4_, and 20 glucose, bubbled with 95% O_2_/5% CO_2_. The pH was adjusted to 7.3–7.4 and the osmolarity to 305 mOsM. We did not suppress GABA_A_ receptor currents, since we were recording at a holding potential of –70 mV close to the chloride reversal potential. All recordings were performed at room temperature. sMSN were identified by their morphology and characteristic electrophysiological properties including negative resting membrane potentials and slow capacitance transients. To confirm the morphology typical for sMSN, we labeled some cells (n = 5) using 200 μM Oregon-green-BAPTA1 (Life Technologies, Carlsbad, USA) and imaged these cells using a confocal microscope (Olympus Fluoview FV1000). For analyses, cells were accepted if the leak current was <100 pA and the resting membrane potential below –70 mV to ensure homogenous recordings.

Glass electrodes (5–8 MΩ) were filled with a solution containing (in mM): 150 potassium gluconate, 10 NaCl, 3 Mg-ATP, 0.3 GTP, 10 HEPES and 0.05 EGTA, adjusted to pH 7.3 and an osmolarity of 305 mOsM. The liquid junction potential of 15 mV was corrected post hoc.

Spontaneous excitatory postsynaptic potentials (sEPSPs) were recorded at –70 mV. We did not apply tetrodotoxin but applied CNQX in some of the recorded neurons to confirm that AMPA receptor activations were the source of spontaneous postsynaptic activity. Spontaneous postsynaptic potentials were analyzed using ClampFit 9 software and the event detection, template search mode (Axon Instruments, Foster City, USA). Templates were generated by averaging 10 characteristic events.

Excitatory sMSN afferents were stimulated with a glass electrode filled with ACSF and placed between the recorded sMSN and the cortex, typically ∼50–100 μm from the cell body using current clamp mode. Stimulus intensity was adjusted to yield large EPSP amplitudes without eliciting an action potential or inducing direct stimulation. Typically, we used 100–200 μA for duration of 0.1 s. Continuous stimulation was performed at a frequency of 0.5 Hz. Following 20 min of baseline stimulation, we applied 3 bursts of 3 s duration and a frequency of 100 Hz separated by 30 s. LTD was then measured for 30 min. Data points were calculated every 30 s by averaging the last 15 EPSPs (0.5 Hz). All recordings were performed using a Multiclamp amplifier (Axon Instruments, Union City, USA), filtered at 2 kHz and digitized at 10 kHz. Acquisition and analysis were performed using custom Clampex 9 software (see above). LTD data were analyzed using a two-tailed Student’s t-test. Data are expressed as mean ± SEM.

## Results

### Transcriptome-wide Striatal Gene Expression Analysis

To identify adaptive gene regulation in response to RGS9 deficiency, striatal gene expression was assayed on a whole transcriptome-scale in 3-month-old male RGS9-deficient and wt mice. RNA was prepared from striatal tissue, reversely transcribed and subjected to Affymetrix microarray hybridization.

To validate the microarray data, we first analyzed relative expression levels of the RGS9 transcript. Surprisingly, the RGS9 transcript was found significantly up-regulated in the striata of RGS9-deficient mice (Table S5 in File S1). These data were confirmed by qPCR (Table 1; Table S4 in File S1). Besides the catalytic RGS domain, RGS9 contains GGL and DEP domains [28]–[29], the latter of which had been substituted in frame by an MC1neopA-cassette to generate the RGS9-deficient mice. Two transcript variants of RGS9 were detected in RGS9-deficient mice, one long including the MC1neopA-cassette and one truncated variant where the MC1neopA-cassette was missing [20]. Elevated transcript concentration of RGS9 detected in striata of RGS9-deficient mice resulted from both transcript variants since RGS9 probe sets and qPCR primer bound to C-terminal regions that were available in both transcript variants. Our Western blot analysis confirmed absence of RGS9 protein in striatal tissue of RGS9-deficient mice [20] (data not shown). This is because the resulting mRNAs did not encode for immunoreactive RGS9 proteins. One can speculate that the up-regulation of the RGS9 transcript variants may be directed towards balancing the lack of RGS9 protein. Indeed, a large fraction of differentially expressed genes belong to the GO category ‘DNA-dependent regulation of transcription’ (Table S2 in File S1). Because the promoter of RGS9 and its regulation is not characterized yet, we did not follow this interesting finding.

**Table 1:** Expression analysis using qPCR of selected transcripts involved in LTD, LTP and Ca^2+^ signaling.

Trancript	gene symbol	qPCR	signal transduction pathway		
adenylate cyclase 3 (AC3)	Adcy3	0.77±0.06** (9)			Ca^2+^
adenylate cyclase 5 (AC5)	Adcy5	0.87±0.42 (6)			Ca^2+^
ATPase, Ca^2+^ transporting, cardiac muscle, slow twitch 2 (SERCA2)	Atp2a2	0.56±0.1** (6)			Ca^2+^
calmodulin 1 (CaM1)	Calm1	0.27±0.12* (6)		LTP	Ca^2+^
Ca^2+^/calmodulin-dependent protein kinase II, beta (CaMK II)	Camk2b	0.74±0.08* (6)		LTP	Ca^2+^
Ca^2+^/calmodulin-dependent protein kinase II gamma (CaMK II)	Camk2g	0.69±0.04*** (6)		LTP	Ca^2+^
Guanine nucleotide binding protein, alpha inhibiting 1 (Giα1)	Gnai1	0.54±0.11* (6)	LTD		
Guanine nucleotide binding protein, alpha inhibiting 2 (Giα2)	Gnai2	0.56±0.11* (6)	LTD		
Guanine nucleotide binding protein, alpha inhibiting 3 (Giα3)	Gnai3	0.45±0.12* (6)	LTD		
Guanine nucleotide binding protein, alpha q polypeptide (Gqα)	Gnaq	0.60±0.12* (6)	LTD	LTP	Ca^2+^
glutamate receptor, ionotropic, AMPA2 (alpha 2) (GluR2)	Gria2	0.66±0.07** (6)	LTD	LTP	
glutamate receptor, metabotropic 5 (mGlu5)	Grm5	0.82±0.07* (9)	LTD	LTP	Ca^2+^
V-Ki-ras2 Kirsten rat sarcoma viral oncogene homolog (K-Ras2)	Kras	0.43±0.14* (6)	LTD	LTP	
mitogen activated protein kinase 3 (ERK1)	Mapk3	0.54±0.1** (6)	LTD	LTP	
Phosphodiesterase 4B, cAMP specific (PDE4b)	Pde4b	0.66±0.12* (6)			
phospholipase C, beta 1 (Plcβ1)	Plcb1	0.53±0.11* (6)	LTD	LTP	Ca^2+^
protein phosphatase 1, regulatory (inhibitor) subunit 1B (DARPP32)	Ppp1r1b	0.76±0.05** (6)			
protein kinase, cAMP dependent, catalytic, beta (PKA)	Prkacb	0.67±0.11* (9)		LTP	Ca^2+^
Protein kinase C, beta 1 (PKC)	Prkcb1	0.51±0.09** (6)	LTD	LTP	Ca^2+^
Rap guanine nucleotide exchange factor (GEF) 3	Rapgef3	0.66±0.1* (9)		LTP	
Regulator of G-protein signaling 9 (RGS9)	Rgs9	1.47±0.2* (6)			
Ryanodine receptor 1, skeletal muscle	Ryr1	0.19±0.04*** (6)	LTD		Ca^2+^

Gene expression data are given as 2^−ΔΔC^_T_ ± SEM with the sample size in parentheses. Additionally, the affiliation to LTD, LTP, and the Ca^2+^-signaling pathway is given for each gene. More microarray and qPCR data including non-changed and other significantly changed signaling components are given in Tables S4 and S5 in File S1. *P≤0.05, **P≤0.01, ***P≤0.001.

Of 45,101 probe sets assayed in the microarray hybridization experiments, 22,962 probe sets, corresponding to 12,714 genes, were detected as present (≥3 present calls in at least one group) using the MAS5 algorithm. To determine the extent of differences in gene expression, we subjected the MAS5-processed microarray data to statistical analysis. We found that 2.6% (365 probe sets, corresponding to 327 genes) were significantly different between wt and RGS9-deficient mice (P≤0.01) (the complete list of differentially expressed transcripts is given on request). We then used the OntoExpress software [22] to identify functional systems that show global differences in gene expression between wt and RGS9-deficient mice (Table S2 in File S1). Differentially expressed transcripts grouped into processes which included transcription, synaptic transmission, posttranslational protein modification like dephosphorylation and palmitoylation, and locomotor behavior (Table S2 in File S1). In a complementary approach, full array data were subjected to gene set enrichment analysis (GSEA) [24] using predefined gene sets obtained from the KEGG database [25]. Genes that are involved in Ca^2+^ signaling and in long-term depression (LTD), a main form of synaptic plasticity, were differentially expressed between wt and RGS9-deficient mice (Table S3 in File S1).

### Expression Analysis of Genes Involved in Ca^2+^ Signaling and Synaptic Plasticity

To verify the differences in gene expression identified by microarray analysis, we performed qPCR for selected transcripts involved in D2R signaling, Ca^2+^ signaling and the two forms of synaptic plasticity, LTD and long-term potentiation (LTP). Verification of microarray data is strongly recommended since different probe set data for one gene can vary in array analyses (see Table S4 in File S1) and microarray and qPCR data do not always show strong correlation [30]–[32]. We therefore considered as differentially expressed only transcripts that revealed similar tendency in both methods and were significantly regulated at least in one of the methods.

Concerning D2R signaling pathway, there was no alteration in D2R transcript concentration (Table S4 in File S1) but the G-protein α_i_ subunits 1–3, the adenylyl cyclase 3 (AC3), the catalytic subunit of protein kinase A (PKA) and phosphodiesterase 4b (PDE4b) were significantly down-regulated in RGS9-deficient mice (Table 1).

**Figure 1:**
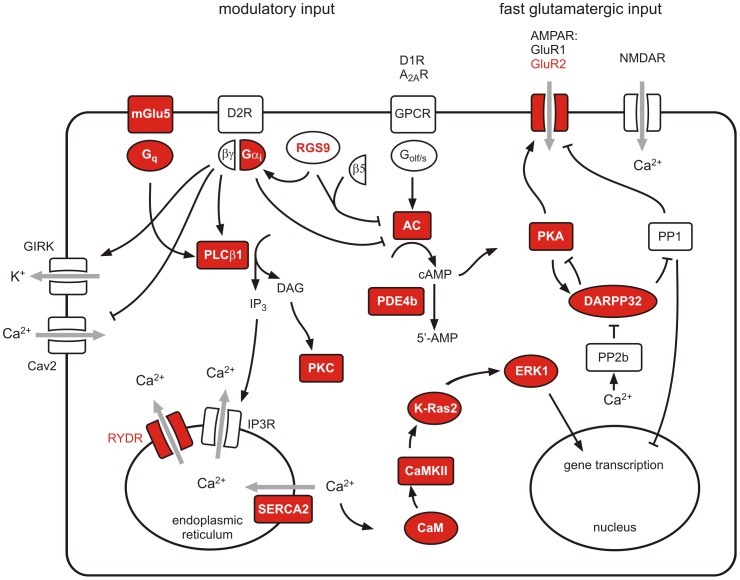
Intracellular localization and functional interaction of selected signaling components expressed in striatum. Transcripts of signaling components that are significantly downregulated in striata of RGS9-deficient mice are shown in red. The full notations of the transcripts are given in Table 1 and Table S4 (in File S1).

Many genes that are involved in Ca^2+^ signaling where down-regulated in striata of RGS9-deficient mice (Table 1) among them the metabotropic glutamate receptor 5 (mGluR5), the ionotropic glutamate receptor subunit α2 (GluR2), Ca^2+^-dependent proteins like calmodulin (CaM1), Ca^2+^/CaM-dependent protein kinase II (CaMK II), phospholipase C (PLCβ1) and a catalytic subunit of protein kinase C (PKC). We also investigated expression levels of genes encoding endoplasmic membrane proteins that regulate the cytoplasmic Ca^2+^ concentration, the ryanodine- (*Ryr1*) and inositol 1,4,5-trisphosphate receptors (*Itpr1*) receptor genes and the most abundant striatal sarco−/endoplasmic reticular Ca^2+^ ATPase (SERCA) gene (*Atp2a2*). While there was no significant alteration in the IP3R1 expression level, Ryr1 and SERCA2 were significantly down-regulated (Table S4 in File S1, Table 1). The intracellular localization and functional interaction of proteins differentially expressed on the transcript level in striata of RGS9-deficient mice is illustrated in Figure 1.

### Expression and Phosphorylation State of Striatal Signaling Proteins

**Figure 2:**
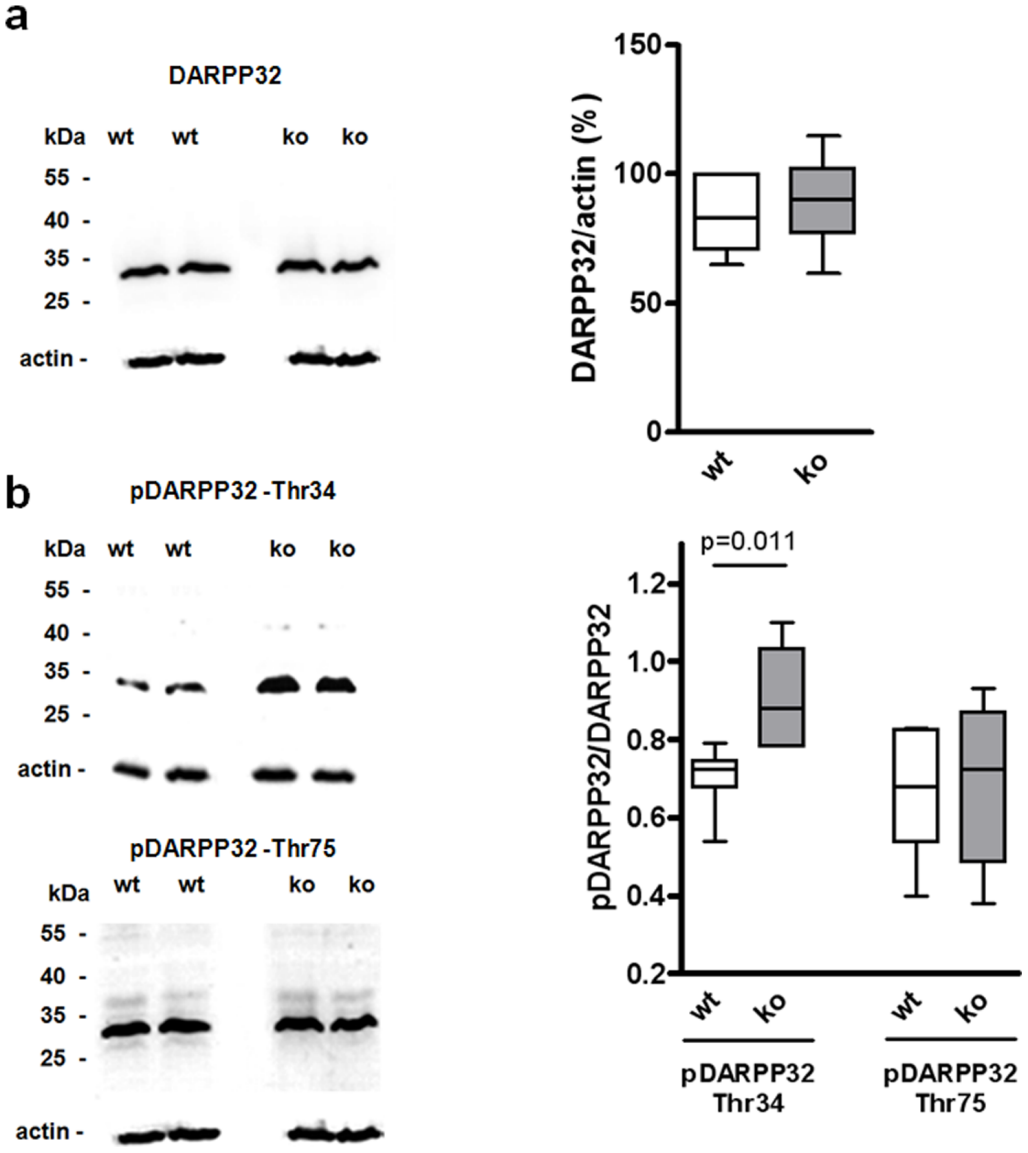
Striatal DARPP32-Thr34 phosphorylation is enhanced in RGS9-deficient mice. (**a**) Total DARPP32 from striatal homogenates of 3-month-old wt and RGS9-deficient mice was measured by Western blot quantification and normalized to actin. (**b**) DARPP32 phosphorylation at Thr34 and Thr75 is given relative to total DARPP32. Representative Western blots are shown, quantification was performed with n = 6–8 per genotype.

To assess the functional relevance of the striatal gene regulation pattern detected in mRNA expression experiments, we subjected striatal homogenates of wt and RGS9-deficient mice to Western blot analyses and quantified selected proteins involved in Ca^2+^ signaling and synaptic plasticity. The dopamine- and cAMP-regulated phosphoprotein of 32 kDa (DARPP32) is a key component in the integration of dopaminergic and glutamatergic signaling and is highly enriched in striatal tissue [33]–[35]. To evaluate differences in RNA expression data from microarray (no regulation) and qPCR (down-regulation in RGS9-deficient mice) DARPP32 protein levels were determined in Western blot analysis revealing no significant difference between both mouse strains (Fig. 2a). DARPP32 activity is regulated by phosphorylation and possesses four functional relevant phosphorylation sites: Thr34, Thr75, Ser102 and Ser137 [36]. Using phosphorylation-specific antibodies, we quantified relative phosphorylation state levels of the most important phosphorylated forms of DARPP32, pDARPP32-Thr34 and pDARPP32-Thr75. Interestingly, phosphorylation of DARPP32 was significantly increased at Thr34, but not at Thr75 (Fig. 2b).

**Figure 3:**
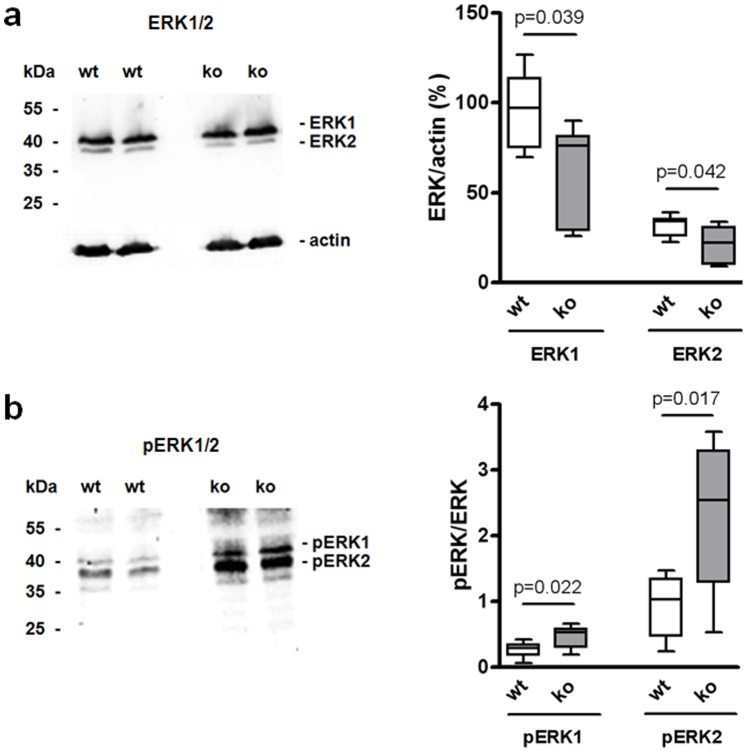
Striatal ERK1 and ERK2 expression are decreased in RGS9-deficient mice but phosphorylation is increased. (**a**) Total ERK1 and ERK2 from striatal homogenates of 3-month-old wt and RGS9-deficient mice was measured by Western blot quantification and normalized to actin. (**b**) Striatal phospho-ERK1/2 relative to total ERK1/2. Representative Western blots are shown, quantification was performed with n = 7 per genotype.

The extracellular signal-regulated kinases 1 and 2 (ERK1 and ERK2) are downstream effectors of dopamine signaling that regulate excitability and glutamatergic transmission in striatal sMSN [37]. The ERK activities are tightly regulated by phosphorylation. Although ERK1 and ERK2 mRNA levels (Table 1, Table S4 in File S1) and protein concentrations (Fig. 3a) were lower in RGS9-deficient mice, ERK1/2 phosphorylation was strongly increased (Fig. 3b) indicating sustained activation.

**Figure 4:**
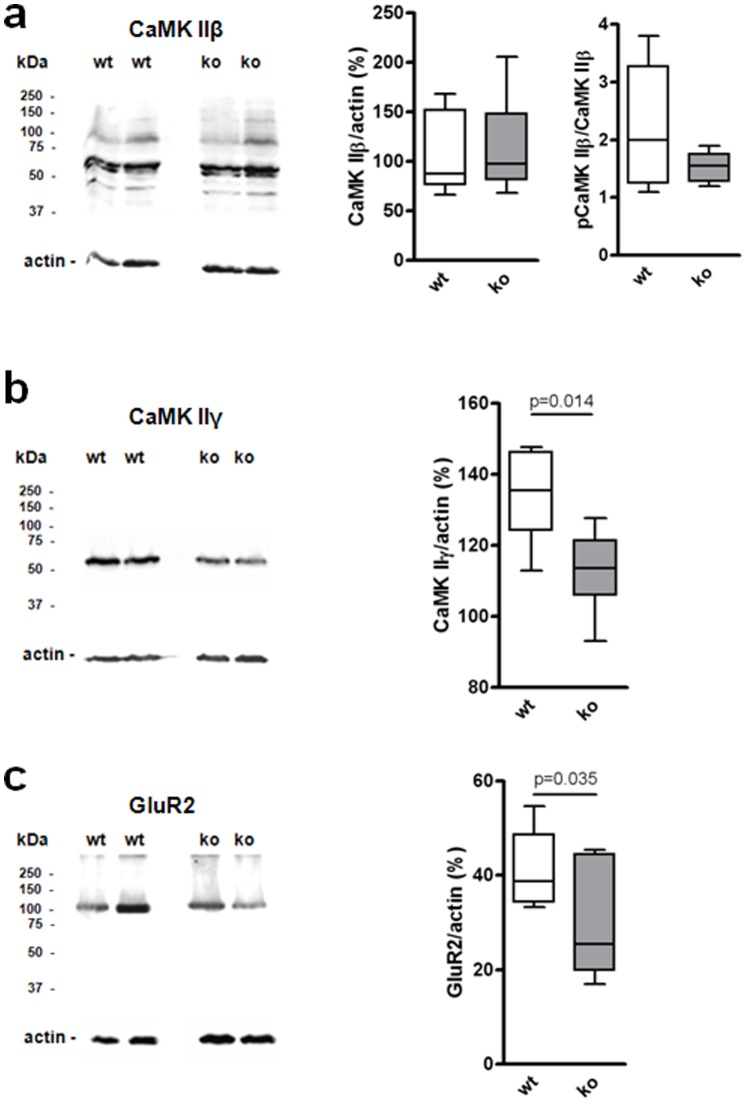
Western blot analyses of key proteins in striata of 3-month-old wt and RGS9-deficient mice. (**a**) CaMK IIβ expression (56 kDa) and phospho-CaMK IIβ expression (60 kDa) were not significantly changed in striata of RGS9-deficient mice. Phospho-CaMK IIβ is given relative to total CaMK IIβ. (**b, c**) CaMK IIγ expression and GluR2 expression were significantly decreased in striata of RGS9-deficient mice. Data were normalized to actin. Representative Western blots are shown, quantification was performed with n = 6 (a, b) and n = 7 (c) per genotype.

Control of glutamatergic transmission is also dependent on Ca^2+^ signaling. Thus, we investigated concentration of Ca^2+^-dependent proteins, e.g. CaMK IIβ and CaMK IIγ (Fig. 4a, b). Furthermore, protein concentration of GluR2, the most abundant striatal AMPA receptor subunit that accounts for Ca^2+^ impermeability of AMPA receptors [38], was determined (Fig. 4c). In agreement with gene expression data, protein concentration of CaMK IIγ and GluR2 were significantly decreased in RGS9-deficient mice. Western blot analysis also revealed a reduced concentration of CaMK IIβ and phospho-CaMK IIβ in RGS9-deficient mice but tendencies were not significant.

### Functional Analysis in Acute Striatal Samples

**Figure 5:**
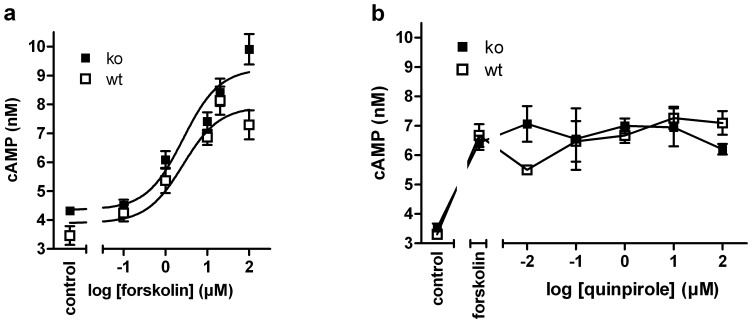
D2R activation does not inhibit forskolin-stimulated cAMP formation in acute striatal samples. (**a**) Forskolin increases cAMP accumulation in acute striatum biopsies from both wt and RGS9-deficient mice with an EC_50_ of 2.6 μM (n = 4). (**b**) Acute striatum samples prestimulated with 10 μM forskolin were incubated with the selective D2R agonist quinpirole at concentrations from 10 nM to 100 μM. No reduction in cAMP accumulation was detected at sub-micromolar concentrations. Similar results were obtained when 60 μM forskolin were used for prestimulation (data not shown).

The hyperphosphorylation of DARPP32-Thr34, that was observed in RGS9-deficient mice (Fig. 2b), implies enhanced basal PKA activity or an imbalance in the activities of the opposing D1R and D2R signaling [36] (Fig. 1). To dissect pathways involved hyperphosphorylation of DARPP32 we assessed basal and stimulated adenylyl cyclase activity in acute striatal samples of wt and RGS9-deficient mice by a label-free AlphaScreen-based cAMP accumulation assay (Fig. 5). Under basal conditions and forskolin stimulation RGS9^−/−^ samples had a tendency toward higher adenylyl cyclase activity, however the tendency was not significant at the individual data point (Fig. 5a). No significant differences between wt and of RGS9^−/−^ striatal samples were seen in quinpirole-induced D2R signaling following forskolin stimulation (Fig. 5b) although the D2R levels appear to be per se below functional detection limits of the assay.

### Analysis of Spontaneous Synaptic Events

**Figure 6:**
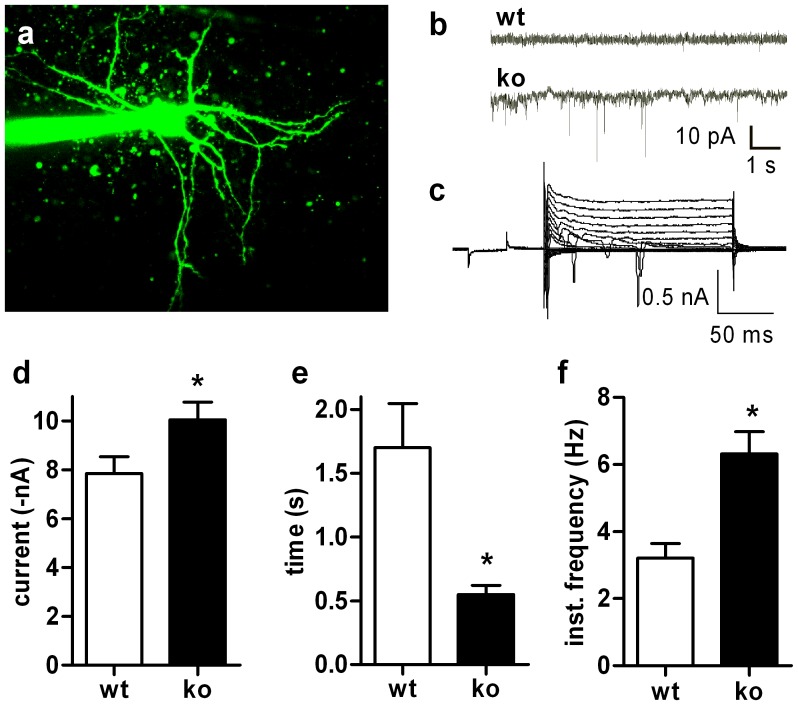
sEPSP in sMSN from wt and RGS9-deficient mice. sEPSP in sMSN from wt and RGS9-deficient mice. (**a**) sMSN with multiple dendrites and a large number of spines, i.e. a typical morphology in wt. (**b**) Characteristic whole-cell currents showing enhanced sEPSP in RGS9^−/−^ cells. (c) Example of voltage clamp traces of RGS9^−/−^ sMSN. (**d–f**) Quantification of amplitudes (**d**), inter-event interval (**e**) and frequency (**f**) of sEPSPs indicating larger and more frequent sEPSPs in RGS9-deficient animals (*P≤0.05, n = 21 (wt) and 18 (ko), respectively).

The expression data (Table 1, Table S4 in File S1, Fig. 4) indicate that GluR2 may be primarily involved in conferring changes in signal transduction induced by the lack of RGS9. Ca^2+^ permeability of AMPA receptors is markedly influenced by subunit composition of these receptors with GluR2 decreasing Ca^2+^ permeability. In fact, overexpression of Ca^2+^-permeable receptors can markedly harm cellular integrity and lead to neurodegeneration [39]. In addition, the ratio of GluR1 to GluR2 also correlates to the amplitude of spontaneous excitatory postsynaptic potentials (sEPSPs) [40]. The significant reduction of GluR2 expression detected in striata of RGS9-deficient mice (Table 1, Fig. 4c) suggests a change in the GluR1:GluR2 ratio and hence a change in the amplitude of sEPSPs. To test this we analyzed sEPSPs in sMSN derived from wt and RGS9-deficient mice (Fig. 6). sEPSPs did not only exhibit larger amplitudes in RGS9-deficient animals (−10.08±0.77 vs −7.85±0.69 pA) but also occurred at a higher frequency (6.31±0.66 vs 3.21±0.44 Hz). All sEPSPs in these cells were completely blocked by CNQX which confirms that these are originated by AMPA receptor activation. We cannot completely exclude that these changes found are exclusively related to postsynaptic AMPA receptors since AMPA receptors are also found presynaptically. However, our expression data strongly suggest that changes in mRNA expression levels mainly derived from sMSN.

### Analysis of Long Term Depression (LTD)

**Figure 7:**
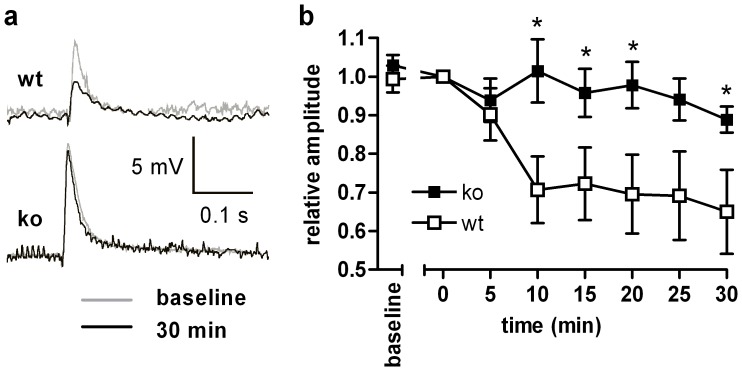
Reduced synaptic plasticity in RGS9-deficient mice. (**a**) EPSPs in sMSN before and 30 min after tetanic stimulation of glutamatergic afferents. (**b**) Normalized amplitudes of evoked EPSPs during the last 5 minutes of baseline stimulation and during the first 30 minutes following tetanic stimulation. Note the expected reduction of amplitudes in wt animals which was not seen in RGS9-deficient animals (*P≤0.05, n = 10 (ko) and 9 (wt), respectively).

Ionotropic glutamate receptors are involved in synaptic plasticity and long term changes following tetanic stimuli. Using Mg^2+^-containing solutions and current clamp mode, LTD, a major type of synaptic plasticity, is well recognized in sMSN and mostly attributed to activation of AMPA receptors. NMDA receptors should not be involved due to voltage and magnesium blocks. LTD is dependent on the expression of GluR2 in cerebellar Purkinje cells [41] and LTD in midbrain dopaminergic neurons depends on the rapid insertion of GluR2 [42]. As GluR2 transcript and protein concentration are significantly reduced in RGS9^−/−^ striata, LTD should be dampened in RGS9^−/−^ compared to wt striata. In fact, when we applied tetanic stimuli and recorded evoked EPSPs over at least 30 min, we observed LTD of ∼10% in RGS9-deficient animals and ∼35% in wt animals (Fig. 7).

## Discussion

Previous studies have employed the RGS9-deficient mouse strain as a genetic model system for potentiated D2R-dependent striatal dopaminergic signal transduction [16]. Indeed, our gene expression data are highly consistent with striatopallidal D2R hyperactivity in RGS9-deficient mice which is counteracted through changes in mRNA expression levels of specific downstream signaling components. Transcript levels of all G-protein α_i_ subunits, the downstream signaling components of G-protein βγ heterodimers, PLCβ1 and the diacylglycerol- (DAG) and Ca^2+^-dependent protein kinase C β1 (PKCβ1) were significantly down-regulated. These findings can be interpreted as compensatory regulation to counteract persistent overactivity of D2R-dependent signaling. Unchanged basal cAMP levels in acute striatum biopsies of wt and ko mice (Fig. 5a) may reflect the effectiveness of these changes at the functional level and/or the minor importance of AC inhibition by striatal D2R signal transduction. Indeed, we did not detect adenylyl cyclase inhibition by the D2R-specific agonist quinpirole in both wt and RGS9-deficient mice (Fig. 5b). The obvious lack of quinpirole-induced cAMP reduction is in agreement with a recent study in rat striatal tissue homogenates [43] suggesting that adenylyl cyclase inhibition is not a significant downstream effect of striatal D2R activation. Relevant D2R-signaling mechanisms in striatopallidal sMSN include activation of PLCβ isoforms [44], inhibition of Cav2 channels [45]–[46] and opening of potassium channels [47]–[48] through interaction with G-protein βγ heterodimers. While the former leads to intracellular Ca^2+^ mobilization, the last two mechanisms result in a dopamine-dependent reduction in neuronal excitability.

There is evidence from our transcriptome data that components of the intracellular D1R/Gs protein/AC signaling cascade (AC 3 and 7, β subunit of PKA) are significantly down-regulated in RGS9-deficient mice. Together with the similar cAMP responses to forskolin (Fig. 5) this may point towards a compensation of increased stimulation via D1R. Secondly, key substrates of PKA that regulate striatal control of motor function include AMPA receptors and Thr34 of DARPP32. In agreement with PKA overactivity, significantly enhanced phosphorylation of DARPP32-Thr34 but not of Thr75 was detected in Western blot experiments (Fig. 2). In DARPP32, Thr34- and Thr75 phosphorylations have mutually antagonistic effects with the phospho-Thr34 form acting as an endogenous inhibitor of protein phosphatase 1. Overactivity of these intracellular effectors and the most abundant striatal AMPA receptor subunit GluR2 is also reflected by down-regulation of the respective transcripts and proteins (Table 1, Fig. 4c).

PLCβ activation is one of the intracellular key events after activation of D2R in striatopallidal sMSN [44]. Inositol trisphosphate release by PLCβ triggers the liberation of Ca^2+^ from the endoplasmic reticulum (Fig. 1). The time course of the intracellular Ca^2+^ signal is further shaped by Ca^2+^-induced Ca^2+^ release from the endoplasmic reticulum, a process that involves ryanodine receptors. Both transcripts Plcb1 and Ryr1, as well as protein kinase C (PKC), a target of the other second messenger released by PLC, diacylglycerol (DAG), were significantly down-regulated in RGS9-deficient mice. In fact, down-regulation of the Ryr1 transcript by a factor of 0.19 was the strongest gene regulation effect observed in RGS9-deficient mice. This is likely to represent an adaptive response to substantially potentiated Ca^2+^ signaling in striatopallidal sMSN. A somewhat puzzling finding is that the ATP2a2 transcript that codes for the most prevalent neuronal sarco/endoplasmic reticulum Ca^2+^-ATPase (SERCA) isoenzyme is also significantly down-regulated. SERCA enzymes participate in the control of [Ca^2+^]_i_ by sequestering Ca^2+^ into the endoplasmic reticulum (Fig. 1). However, in a recent study, profound loss of striatal SERCA activity was also found in an animal model of excitotoxicity that is associated with excessive Ca^2+^ influx [49]. Further stimulation of PLCβ isoenzymes comes from G_q/11_ protein-coupled receptors, particularly class I metabotropic glutamate receptors. Both mGluR5 and the G-protein α_q_ subunit were also significantly down-regulated in RGS9^−/−^ striata (Table 1). Taken together, the described effects may result in exaggerated Ca^2+^ release in striatopallidal sMSN. This finding would also be consistent with the above mentioned circuitry-based increased glutamatergic stimulation of RGS9^−/−^ striata.

The intracellular Ca^2+^ concentration ([Ca^2+^]_i_) controls a plethora of cellular effects, an action that is in many cases mediated by the intracellular Ca^2+^ sensor calmodulin (CaM). In sMSN, this includes regulation of gene transcription, cellular excitability and long-term modulation of ionotropic glutamatergic transmission. Many processes that control synaptic transmission including AMPA receptor function [50] and synaptic delivery [51] are subject to regulation by the Ca^2+^/CaM-dependent kinase II (CaMK II). Both, the CaM and CaMK II (isoforms β and γ) transcripts were significantly down-regulated (Table 1) indicating sustained activity of this pathway. Enhanced synaptic AMPA receptor activity is also functionally reflected in the significantly increased amplitude and frequency of sEPSP in voltage-clamp experiments (Fig. 6).

AMPA receptors are heterotetrameric complexes in which the presence of the GluR2 subunit defines the endogenous impermeability to Ca^2+^ [38]. There is, however, increasing evidence that Ca^2+^-permeable forms of AMPA receptors may participate in striatal plasticity [52]. Both qPCR and Western blot experiments demonstrated significantly reduced striatal expression of GluR2 in RGS9-deficient mice (Table1; Fig. 4c). This favors the formation of Ca^2+^-permeable AMPA receptors that may contribute to excessive Ca^2+^ signaling and altered long-term synaptic plasticity in sMSN.

LTD is the most prominent form of synaptic plasticity in the dorsal striatum. Studies in transgenic mice in which direct and indirect pathway sMSN were labeled by selective GFP expression revealed that high-frequency stimulation-induced LTD is a specific feature of D2R-expressing indirect pathway sMSN [53]. There is evidence that the underlying mechanism depends on the postsynaptic release of endocannabinoids in a process that involves D2R stimulation, L-type Ca^2+^ channels and group I metabotropic glutamate receptors (mGluR1, mGluR5) in a coordinated way [54]–[57]. The striking reduction in synaptic LTD in RGS9^−/−^ sMSN (Fig. 7) may be the result of mGluR5 down-regulation or attenuation of the intracellular D2R signaling cascade. The marked disturbances in working memory that have been previously described in RGS9-deficient animals [19] may be a behavioral correlate to this finding.

We also found an increased ERK1/2 phosphorylation (Fig. 3). However, this does not result from enhanced Ca^2+^ signaling and CaMK II overactivity because Western blot analysis revealing no significant difference in phosphorylation state of CaMK IIβ in RGS9-deficient mice (Fig. 4a). A number of studies indicate that ERK1/2 phosphorylation underlies striatal regulation of gene expression and long-term synaptic plasticity [58]. In a mouse model of drug-induced dyskinesia, sensitized cAMP/PKA signaling activated ERK1/2 [59], a pathway that is confined to D1R-expressing sMSN [60]–[61].

It remains unclear, how constitutively potentiated D2R activity in RGS9-deficient mice modulates D1R-dependent transmission and whether this crosstalk occurs in the same sMSN. However, a significant subset of MSN (20–50% depending on the striatal area) but not all coexpress both D1R and D2R [62]–[64]. In a previous study that analyzed striatal dopaminergic signal transduction in mice deficient for the G-protein α_o_ splice variant, attenuation of D2R signaling resulted in concomitant down-regulation of D1R-signaling components and diminished behavioral sensitization in response to cocaine [65]. Enhancement of D1R transmission in RGS9-deficient mice may thus be an analogous compensatory response on a functional level to D2R overactivity. We cannot answer whether these compensatory responses involve alterations of the activity of the basal ganglia loop based on the data presented. However, the basic model relating to direct and indirect signaling pathways in the basal ganglia would predict, that increased D2R signaling of sMSN lead to decreased activity of subthalamic nucleus and medial pallidal (GPi) output neurons and subsequent overactivity of cortical motor neurons [66], which in turn would overstimulate sMSN. Our present data provide at least some evidence that such glutamatergic overstimulation may share alterations of downstream signaling molecules such as DARPP32 and ERK.

In summary, RGS9-deficient mice show significant changes in striatal function including enhanced DARPP32 and ERK phosphorylation that are characteristically found in L-DOPA-induced dyskinesia [59], [67]. In this study, we have shown that striata of RGS9-deficient mice present changes in transcript levels of Ca^2+^ signaling components that may counteract D2R overactivity. Thus, prolonged dopamine depletion that releases the pressure on gene regulation unmasks dyskinesia in these animals. By analyzing specific gene expression in RGS9-deficient mice, we have identified a number of critical signaling components that may represent novel targets in antidyskinetic therapy.

## Supporting Information

File S1 Contains the following files. **Table S1. Primers used for qPCR.** (fwd = forward, rev = reverse). **Table S2. OntoExpress analysis of genes differentially regulated in RGS9-deficient mice.** MAS5-processed microarray data were filtered for probe sets ≥3 present calls. Probe sets that were significantly regulated (**P≤0.01) between wt and RGS9-deficient mice were subjected to gene ontology analysis. The table shows the statistically regulated biological processes ranked by the total number of genes regulated. **Table S3.** **Gene Set Enrichment Analysis (GSEA) of differentially expressed genes in RGS9-deficient mice and wild-type mice. Table S4.** **Expression analysis of selected genes by qPCR.** The table shows all transcripts the concentration of which were determined by qPCR in striata of wildtype and RGS9-deficient mice. Gene expression data are given as 2^−ΔΔC^_T_ ± SEM with the sample size in parentheses. Furthermore, microarray data and the affiliation to LTD, LTP and/or Ca^2+^ signaling pathway are listed for each transcript. *P≤0.05, **P≤0.01, ***P≤0.001. **Table S5. Microarray expression analysis of striata of RGS9-deficient mice.** The table shows all significant regulated transcripts with a fold-change ≤0.5 or ≥1.5. *P≤0.05, **P≤0.01, ***P≤0.001.
(DOC)
