# Supplementary material for: Adaptive Gene Regulation in the Striatum of RGS9-Deficient Mice
Source: PLoS One. 2014 Mar 24;9(3):e92605. doi: 10.1371/journal.pone.0092605 (PMC3963927; doi:10.1371/journal.pone.0092605)
Supplement: File S1 — Contains the following files. Table S1. Primers used for qPCR. (fwd = forward, rev = reverse). Table S2. OntoExpress analysis of genes differentially regulated in RGS9-deficient mice. MAS5-processed microarray data were filtered for probe sets ≥3 present calls. Probe sets that were significantly regulated (**P≤0.01) between wt and RGS9-deficient mice were subjected to gene ontology analysis. The table shows the statistically regulated biological processes ranked by the total number of genes regulated. Table S3. Gene Set Enrichment Analysis (GSEA) of differentially expressed genes in RGS9-deficient mice and wild-type mice. Table S4. Expression analysis of selected genes by qPCR. The table shows all transcripts the concentration of which were determined by qPCR in striata of wildtype and RGS9-deficient mice. Gene expression data are given as 2−ΔΔC T ± SEM with the sample size in parentheses. Furthermore, microarray data and the affiliation to LTD, LTP and/or Ca2+ signaling pathway are listed for each transcript. *P≤0.05, **P≤0.01, ***P≤0.001. Table S5. Microarray expression analysis of striata of RGS9-deficient mice. The table shows all significant regulated transcripts with a fold-change ≤0.5 or ≥1.5. *P≤0.05, **P≤0.01, ***P≤0.001. (DOC) [file pone.0092605.s001.doc]

# Supplements

**Adaptive gene regulation in the striatum of**

**RGS9-deficient mice**

**Kathy Busse^1,2#^, Rainer Strotmann^2^, Karl Strecker^1^, Florian Wegner^1,3^*, Vasudharani Devanathan^4^, Antje Gohla^5^, Torsten Schöneberg^2#^, and Johannes Schwarz^1,3,6^**

^1^Dept. of Neurology, Medical Faculty, University of Leipzig, Germany

^2^Institute of Biochemistry, Medical Faculty, University of Leipzig, Germany

^3^Translational Centre of Regenerative Medicine (TRM), University of Leipzig, Germany

^4^Institute of Pharmacology and Toxicology, Eberhard Karls University of Tübingen, Germany

^5^Rudolf Virchow Centre of Experimental Biomedicine, Julius Maximilians University of Würzburg

^6^Dept. of Neurology, Technical University of Munich, Germany

*current address: Dept. of Neurology, Hannover Medical School, Germany

^#^Address correspondence to: Kathy Busse, Dept. of Neurology, and Torsten Schöneberg, Institute of Biochemistry, Medical Faculty, University of Leipzig, Johannisallee 30, 04103 Leipzig, Germany, Tel.: +49-341-9725874, Fax: +49-341-9725878, Email: kathy.busse@medizin.uni-leipzig.de, schoberg@medizin.uni-leipzig.de

**Table S1.**

| **transcript** | **gene symbol** | **direction** | **5' → 3' sequence** |
| --- | --- | --- | --- |
| actinin, alpha 1 | Actn1 | fwd | TCC AGG CCT TCA TCG ACT TT |
|  |  | rev | CGC AGC TCA TCC TCT GTG AT |
| actinin alpha 2 | Actn2 | fwd | CTT CCG GAT TCT GGC TTC TG |
|  |  | rev | ATG GGG GCA TTC TCT TGA TG |
| actinin alpha 4 | Actn4 | fwd | TCA TCG CCT CCT TCA AGG TC |
|  |  | rev | ATG CAG TAC TCG GCC TGG TC |
| adenylate cyclase 3 (AC3) | Adcy3 | fwd | CAT GAA GGA CAC GCT CAC AA |
|  |  | rev | CTG GCC ACA TTG ACC GTA TT |
| adenylate cyclase 5 (AC5) | Adcy5 | fwd | GCT AGA GGC CAA CAA TGA GG |
|  |  | rev | GCC ATG TAG GTG CTG CCT AT |
| adenylate cyclase 7 (AC7) | Adcy7 | fwd | GGA CTC TGG CCT GTG AAC TG |
|  |  | rev | TCG GAA TGC TCT GGA GCT AA |
| ATPase, Ca++ transporting, cardiac muscle, slow twitch 2 (SERCA 2) | Atp2a2 | fwd | CAG AAG CCA TCA GCC AAG TC |
|  |  | rev | GGG CCC ATT AGA AAG CAT GT |
| Braf transforming gene | Braf | fwd | TTT TCC TGG CAG GCT GAG TA |
|  |  | rev | ACA CAC CCC TCA TGT GCT TC |
| Calcium channel, voltage-dependent P/Q type, alpha 1A subunit (Cav2.1) | Cacna1a | fwd | GTA CCG CCT GAG GAG AAC CA |
|  |  | rev | TGC TCA GAT CTG TCC CCA AA |
| calbindin-28K | Calb1 | fwd | TCC TCT GCT GGT TAT TGG TGA |
|  |  | rev | CCC AAA CCT GCA TTA GGT GA |
| calmodulin 1 (CaM1) | Calm1 | fwd | GGA ATC CTG CAT GAG AAC CA |
|  |  | rev | GAT GCC AGA AGG CTG AAG TG |
| calmodulin 2 | Calm2 | fwd | GAT GAC AAA CCT TGG CGA GA |
|  |  | rev | TTC ACT TCG CTG TCA TCA TTT G |
| calmodulin 3 | Calm3 | fwd | AGG CCG ACA TTG ATG GAG AT |
|  |  | rev | AGA GAG GAG AGC GCG AGA AG |
| Calcium/calmodulin-dependent protein kinase II, beta (CaMK II) | Camk2b | fwd | TTG GGG TAC TTG TCC CTT CC |
|  |  | rev | CTG TTC CCA GTA TCC GTC CA |
| Calcium/calmodulin-dependent protein kinase II, delta (CaMK II) | Camk2d | fwd | GCC CCA GTT TTC TTC TCG TC |
|  |  | rev | GTG GAG GTA ACG CTG ATG GA |
| Calcium/calmodulin-dependent protein kinase II, gamma (CaMK II) | Camk2g | fwd | TGG TGC TGC AGA AGT GTG TC |
|  |  | rev | CGT GCA TTG TGC ATC CTC TA |
| Cholinergic receptor, muscarinic 4 | Chrm4 | fwd | GCT GCC TTC TAC CTG CCT GT |
|  |  | rev | GGC TCT TGA GGA AAG CCA GA |
| DEP domain containing 6 | Depdc6 | fwd | CTG GGG CTT TGT GGT ACG AG |
|  |  | rev | GGC CAT TGA CAG AGA CGA CA |
| dopamine receptor D1A | Drd1a | fwd | GAC AGA TGC ATT GTT GAT AAT GGT TT |
|  |  | rev | CTC TGA TTT AAC ATT GGT TCG GTC T |
| dopamine receptor 2 (D2R) | Drd2 | fwd | ACC CGG ACC TCC CTT AAG AC |
|  |  | rev | GCA GCC AGC AGA TGA TGA AC |
| gamma-aminobutyric acid (GABA-A) receptor, subunit alpha 2 | Gabra2 | fwd | CAA AAA GAG GAT GGG CTT GG |
|  |  | rev | AGC TAC CGC ATA GGC GTT GT |
| guanine nucleotide binding protein alpha inhibiting 1 (Giα1) | Gnai1 | fwd | CGT CCA TCA TCC TTT TCC TCA |
|  |  | rev | GGC CGC TTC TTC ATA TGT GTT |
| guanine nucleotide binding protein alpha inhibiting 2 (Giα2) | Gnai2 | fwd | CCT GAA GGA CTG TGG CCT CT |
|  |  | rev | CAG GCA GGG AAC ATG GTC TT |
| guanine nucleotide binding protein alpha inhibiting 3 (Giα3) | Gnai3 | fwd | TGG GCT TTA TTG AGA GGA TGG |
|  |  | rev | GTG CTG CTG ACC CAA GAG AC |
| guanine nucleotide binding protein alpha o (Goα) | Gnao1 | fwd | CCG TCA CCG ACA TCA TCA TT |
|  |  | rev | ACA AAG GCC AAA GGT CAT GC |
| guanine nucleotide binding protein alpha q polypeptide (Gqα) | Gnaq | fwd | ATG TCG CCC TTT TGT GTG TG |
|  |  | rev | GGC GTG TGT TTT CCC TTT GT |
| GNAS (guanine nucleotide binding protein alpha stimulating complex locus (Gsα) | Gnas | fwd | GCA AGA CCT GCT TGC TGA GA |
|  |  | rev | GTC ACG CGT GGG TCC TCT |
| guanine nucleotide binding protein, beta 5(Gβ5) | Gnb5 | fwd | GGG ATG TGG AAA GTG GAC AG |
|  |  | rev | TCT TGT CAC AAC CCC CAG A |
| glutamate receptor, ionotropic, AMPA1 (alpha 1) (GluR1) | Gria1 | fwd | CGG ACA ACC ACC ATC TCT GT |
|  |  | rev | CTG CAG GAC ATT GCT TCA CA |
| glutamate receptor, ionotropic, AMPA2 (alpha 2) (GluR2) | Gria2 | fwd | GTA TGT GCG TGC TGA TGT GG |
|  |  | rev | GCA GTG GAC CCA GGC TAG TT |
| glutamate receptor, ionotropic, AMPA3 (alpha 3) (GluR3) | Gria3 | fwd | AGC ATC ACT CCC AGG TCC TT |
|  |  | rev | TGA CGT CAA CAG CAG GTT TG |
| glutamate receptor, ionotropic, NMDA2C (epsilon 3) (NR2C) | Grin2c | fwd | GGG AGC ACC TGG TCT ACT GG |
|  |  | rev | GGC TCT GTA CCC CGT TGA AG |
| glutamate receptor, metabotropic 1 (mGlu1) | Grm1 | fwd | TGG TCT GAA CCA GGT GGA AG |
|  |  | rev | TAC AGG CCG TCT CAT TGG TC |
| Glutamate receptor, metabotropic 5 (mGlu5) | Grm5 | fwd | TGC CTG CCT CTA CCT GAA AA |
|  |  | rev | GAC ACT AAC CCC AGG CAA ATG |
| guanylate cyclase 1, soluble, alpha 3 | Gucy1a3 | fwd | TCT AAC CCA AGC GGT GTG AA |
|  |  | rev | TCC ATT TGC CCC AGG TGT GT |
| inositol 1,4,5-triphosphate receptor 1 (IP3R) | Itpr1 | fwd | GGG AAC AGA ACG AGC TGA GG |
|  |  | rev | GGC CGA TTC TTT GTT TCT GC |
| potassium inwardly-rectifying channel, subfamily J, member 10 | Kcnj10 | fwd | CAT CCG TCG AGG TTT GAT GA |
|  |  | rev | AAC TTG TTC AGC GGC AGG TT |
| potassium inwardly-rectifying channel, subfamily J, member 3 | Kcnj3 | fwd | AGC GAA GCA TGC AAA CTG AA |
|  |  | rev | AAC GAT GAC CCC AAA GCA CT |
| Potassium inwardly-rectifying channel, subfamily J, member 6 | Kcnj6 | fwd | TTC CGC CTG CAT GTG TGT AT |
|  |  | rev | TCC CAG AGC CTA CTG TAC CTC AA |
| potassium inwardly-rectifying channel, subfamily J, member 9 | Kcnj9 | fwd | GGG CAT TAG CAA CAG GAA GC |
|  |  | rev | CGT CCC GGA CCT ACT GAG TC |
| v-Ki-ras2 Kirsten rat sarcoma viral oncogene homolog (K-Ras2) | Kras | fwd | TTG GGA TGG TGG TAG GCA TT |
|  |  | rev | AAC ACA GCA CAC AGC CCA CT |
| mitogen-activated protein kinase kinase 2 | Map2k2 | fwd | CCC AGT GGT GTG TTC AGC TC |
|  |  | rev | CGC TTG ATG AAG GCG TGG TT |
| mitogen-activated protein kinase 2 | Mapk2 | fwd | TTG AAG TTG AAC AGG CTC TGG |
|  |  | rev | TCG TCC AAC TCC ATG TCA AA |
| mitogen-activated protein kinase 3 (ERK1) | Mapk3 | fwd | AGA CAC CCC TGT CCT TTT GG |
|  |  | rev | GGG TCT GGG TTG AGC AAA GT |
| phosphodiesterase 1A, calmodulin-dependent | Pde1a | fwd | TGG TCA TGT TTT GCC TGA GC |
|  |  | rev | TTC ACG AAG TGT CGC AAT CC |
| phosphodiesterase 4B, cAMP specific (PDE4b) | Pde4b | fwd | AGT TCT GCC CAA CCC TTT GA |
|  |  | rev | TGC TCC TAG CCC CTA CCA AA |
| phosphodiesterase 7B | Pde7b | fwd | CCT TGA ACA GAA GTT TGA ACT G |
|  |  | rev | AGC GGC TCC ACG ATG TAA GT |
| phosphodiesterase 8B | Pde8b | fwd | CCT TGG ACC TGT GCA TTG AA |
|  |  | rev | AAC ACT GGC ATC ACC ACA GG |
| phospholipase A2 receptor 1 | Pla2r1 | fwd | CCG GAA GGA ATA TGG CAC TT |
|  |  | rev | GGA ACG ATG CTG TGG CTT AG |
| phospholipase C, beta 1 (Plcβ1) | Plcb1 | fwd | AAG TGG TGA GCA CCG TCC TT |
|  |  | rev | TCC CTT TCA TGG CTT CCT GT |
| protein phosphatase 1, regulatory (inhibitor) subunit 12A (PP1) | Ppp1r12a | fwd | GTC AGC TCA ACA GGC CAA AC |
|  |  | rev | GCC GTC GTT CTC TGA TTG AC |
| protein phosphatase 1, regulatory (inhibitor) subunit 1B (DARPP32) | Ppp1r1b | fwd | GTT GAG TCC CCA ACC CTC TC |
|  |  | rev | ACC CAG CAG GAA TGG GAT AC |
| protein phosphatase 2, regulatory subunit A (PR 65), alpha isoform (PP2A) | Ppp2r1a | fwd | CCA TCC TGG AGA AGC TGA CC |
|  |  | rev | TTC CAG CAT CAG GCA AGA GA |
| protein phosphatase 3, catalytic subunit, beta isoform (PP2B) | Ppp3cb | fwd | CAG GGA TGT TGC CTA GTG GA |
|  |  | rev | TCC AAA CCC TTT GCC TCT TC |
| protein phosphatase 3, regulatory subunit B, alpha isoform (calcineurin B, type I) (PP2B) | Ppp3r1 | fwd | AAG GGT GGG TTG AAT CAA GG |
|  |  | rev | CAT GCA GAG ACA CCA AGC AC |
| protein phosphatase 3, regulatory subunit B, alpha isoform (calcineurin B, type II) (PP2B) | Ppp3r2 | fwd | GGG AGT GGG AAT CCA CAG AT |
|  |  | rev | GGG GTT CCT ATT CCT TGT GG |
| protein kinase, cAMP dependent, catalytic, alpha (PKA) | Prkaca | fwd | AAC GGG GTC AAT GAC ATC AA |
|  |  | rev | CCA GGG CCT TTA AAC TTT GG |
| protein kinase, cAMP dependent, catalytic, beta (PKA) | Prkacb | fwd | AGG GTA TCC CGC TGT GAA GT |
|  |  | rev | CAG TGA GGG AGG AAG GAA GG |
| protein kinase C, beta 1 (PKC) | Prkcb1 | fwd | GGG CTT TCT GAC CAC TCA GG |
|  |  | rev | CCA AAT GGA AAG CGA ACA CA |
| protein kinase C, iota | Prkci | fwd | AGC AGT GAG AAC CCC CAT TG |
|  |  | rev | GCT CCC TCG CAT TTA TGT CC |
| protein kinase, cGMP-dependent, type I | Prkg1 | fwd | GCA CCT TGA CAC CTC CCA TA |
|  |  | rev | GTC ATC AGG TGG TGG CTC AT |
| RAS related protein 1b | Rap1b | fwd | CGC AAA AAG TCA TCG TGT CA |
|  |  | rev | ACT GCT GAA TTG GGC AAC AG |
| Rap guanine nucleotide exchange factor (GEF) 3 | Rapgef3 | fwd | CAC TCA GAA GCC GAG TGT CC |
|  |  | rev | CGC TGG TTG TCA ATG ACC TT |
| regulator of G-protein signaling 4 | Rgs4 | fwd | GTT CAC CCC ATC CCA AAC AC |
|  |  | rev | GCC TTG AAA AGC TCC AAG CA |
| regulator of G-protein signaling 9 (RGS9) | RGS9 | fwd | CTC TGT GTC ACA CGG GAA GG |
|  |  | rev | AGG CAG GTC CCA CTG TCT GT |
| Regulator of G-protein signaling 9 binding protein (RGS9BP) | RGS9bp | fwd | AGC AAA CTC AGG GAA ACG ATG |
|  |  | rev | CGT CTT CTG ACC ATG GGG TAG |
| ryanodine receptor 1, skeletal muscle | Ryr1 | fwd | GGA GCT CCG AGA CCA ACA AG |
|  |  | rev | CAT GTG GGG TTG TGT CGA AG |
| ryanodine receptor 2, cardiac | Ryr2 | fwd | AGG AAC ACA ACC TGG CCA AC |
|  |  | rev | AGT CCC CTG CTG GGA AAA AT |
| ryanodine receptor 3 | Ryr3 | fwd | GAC ACG ACC CCT CAT GGT TT |
|  |  | rev | CCT GGC CCG TAT GTT CTG TT |

**Table S2.**

| **biological process** | **fraction of significant genes** | **total** | **P-value** |
| --- | --- | --- | --- |
| regulation of transcription, DNA-dependent | 12.05 % | 44 | 0.0054** |
| synaptic transmission | 2.74 % | 10 | 0.0038** |
| small GTPase mediated signal transduction | 2.74 % | 10 | 0.0083** |
| transmembrane receptor protein tyrosine kinase signaling pathway | 2.19 % | 8 | 0.0038** |
| dephosphorylation | 2.19 % | 8 | 0.0043** |
| adult locomotory behavior | 1.92 % | 7 | 0.0010** |
| regulation of phosphorylation | 1.37 % | 5 | 0.0056** |
| neurotransmitter secretion | 1.37 % | 5 | 0.0018** |
| axon guidance | 1.37 % | 5 | 0.0028** |
| neuron migration | 1.10 % | 4 | 0.0090** |
| protein palmitoylation | 0.82 % | 3 | 0.0043** |
| thymus development | 0.82 % | 3 | 0.0018** |
| neurological control of breathing | 0.82 % | 3 | 0.0043** |
| negative regulation of heart contraction | 0.82 % | 3 | 0.0013** |
| response to pain | 0.55 % | 2 | 0.0072** |
| eye photoreceptor cell development | 0.55 % | 2 | 0.0056** |

**Table S3.**

Enrichment in phenotype: KO (5 samples)

•8 / 24 gene sets are upregulated in phenotype KO

•0 gene sets are significant at FDR < 25%

•0 gene sets are significantly enriched at nominal pvalue < 1%

•0 gene sets are significantly enriched at nominal pvalue < 5%

| **Gene set** | **SIZE** | **ES** | **NES** | **NOM p-val** | **FDR q-val** |
| --- | --- | --- | --- | --- | --- |
| MAPK SIGNALLING PATHWAY | 167 | 0.2731769 | 1.3035432 | 0.0945946 | 0.81694734 |
| EICOSANOIDSYNTHESIS | 35 | 0.31676346 | 1.2002461 | 0.12692308 | 0.716518 |
| APOTOSIS | 43 | 0.28176534 | 1.0753151 | 0.3553719 | 0.83958787 |
| INSULIN SIGNALLING PATHWAY | 141 | 0.1972665 | 0.9734315 | 0.5714286 | 0.92444897 |
| JAK STAT SIGNALLING PATHWAY | 20 | 0.29456037 | 0.8660127 | 0.656 | 1 |
| GEN MAPP G-PROTEIN SIGNALING PATHWAY | 77 | 0.20210244 | 0.83848983 | 0.74380165 | 0.91794604 |
| ABC TRANSPORTER | 26 | 0.24820666 | 0.8226416 | 0.8008658 | 0.8204633 |
| ADIPOCYTOKINE SIGNALLING | 44 | 0.20554493 | 0.7977977 | 0.7824268 | 0.7518477 |

Enrichment in phenotype: WT (5 samples)

•16 / 24 gene sets are upregulated in phenotype WT

•2 gene sets are significantly enriched at FDR < 25%

•1 gene sets are significantly enriched at nominal pvalue < 1%

•2 gene sets are significantly enriched at nominal pvalue < 5%

| **Gene set** | **SIZE** | **ES** | **NES** | **NOM p-val** | **FDR q-val** |
| --- | --- | --- | --- | --- | --- |
| LONG TERM DEPRESSION | 53 | -0.4657629 | -1.6002972 | **0.00847458** | **0.14638953** |
| CALCIUM SIGNALLING PATHWAY | 102 | -0.33128104 | -1.4690137 | **0.02419355** | 0.26746547 |
| LONG TERM POTENTIATION | 55 | -0.36409876 | -1.4301012 | 0.07228915 | **0.23358075** |
| NEUROACTIVE LIGAND REZEPTOR INTERACTION | 101 | -0.41187266 | -1.3467276 | **0.05063291** | 0.330351 |
| WNT SIGNALLING PATHWAY | 93 | -0.29788592 | -1.3215446 | 0.08438819 | 0.3191105 |
| PPAR SIGNALLING PATHWAY | 45 | -0.386313 | -1.22381 | 0.19607843 | 0.42176828 |
| GAP JUNCTION | 27 | -0.32005146 | -1.0287179 | 0.43122676 | 0.848497 |
| REGULATION OF ACTIN CYTOSKELLETON | 136 | -0.24247631 | -0.9960135 | 0.5020243 | 0.83293575 |
| ARACHIDONIC ACID METABOLISM | 19 | -0.33462206 | -0.9683669 | 0.49590164 | 0.82748085 |
| TYROSIN METABOLISM | 31 | -0.31642225 | -0.96618867 | 0.4612245 | 0.7494098 |
| CELL CYCLE | 79 | -0.24932659 | -0.9590549 | 0.464 | 0.70033157 |
| PROSTAGLANDIN SYNTHESIS REGULATION | 18 | -0.3638861 | -0.95168537 | 0.526971 | 0.65522754 |
| TIGHT JUNCTION | 81 | -0.21662022 | -0.9265906 | 0.61290324 | 0.65803117 |
| ERBB SIGNALLING PATHWAY | 33 | -0.2574551 | -0.9025103 | 0.60408163 | 0.65154266 |
| CELL HOMOESTASIS | 62 | -0.243159 | -0.8592925 | 0.677686 | 0.6961489 |
| MTOR SIGNALLING PATHWAY | 25 | -0.2417206 | -0.7340023 | 0.8097166 | 0.8553261 |

**Table S4:**

| **transcript** | **gene symbol** | **Probe Set ID** | **microarray** | **qPCR** | **signaling pathway** | | |
| --- | --- | --- | --- | --- | --- | --- | --- |
| actinin, alpha 1 | Actn1 | 1427385_s_at | 1.39 ± 0.06* | 0.98 ± 0.25 (6) |  |  |  |
|  |  | 1428585_at | 1.17 ± 0.02* |  |  |  |  |
|  |  | 1452415_at | 1.16 ± 0.05 |  |  |  |  |
| actinin alpha 2 | Actn2 | 1448327_at | 1.03 ± 0.03 | 0.81 ± 0.14 (6) |  |  |  |
|  |  | 1456968_at | 1.37 ± 0.18 |  |  |  |  |
| actinin alpha 4 | Actn4 | 1423449_a_at | 1.01 ± 0.02 | 0.95 ± 0.2 (6) |  |  |  |
|  |  | 1444258_at | 1.45 ± 0.21 |  |  |  |  |
| adenylate cyclase 3 (AC3) | Adcy3 | 1421959_s_at | 1.18 ± 0.03* | 0.77 ± 0.06** (9) | Ca^2+^ |  |  |
|  |  | 1421960_at | 1.04 ± 0.03 |  |  |  |  |
|  |  | 1436928_s_at | 1.03 ± 0.06 |  |  |  |  |
|  |  | 1436929_x_at | 0.96 ± 0.06 |  |  |  |  |
| adenylate cyclase 5 (AC5) | Adcy5 | 1447696_x_at | 1.06 ± 0.04 | 0.87 ± 0.42 (6) |  |  |  |
|  |  | 1455296_at | 1.03 ± 0.02 |  |  |  |  |
| adenylate cyclase 7 (AC7) | Adcy7 | 1456307_s_at | 1.43 ± 0.08* | 0.65 ± 0.11* (9) | Ca^2+^ |  |  |
| ATPase, Ca^2+^- transporting, cardiac muscle, slow twitch 2 (SERCA2) | Atp2a2 | 1416551_at | 1.03 ± 0.01 | 0.56 ± 0.1** (6) | Ca^2+^ |  |  |
|  |  | 1427250_at | 1.01 ± 0.01 |  |  |  |  |
|  |  | 1427251_at | 1.36 ± 0.03*** |  |  |  |  |
|  |  | 1437797_at | 1.7 ± 0.21 |  |  |  |  |
|  |  | 1443551_at | 1.56 ± 1.17 |  |  |  |  |
|  |  | 1452363_a_at | 1.07 ± 0.02 |  |  |  |  |
| Braf transforming gene gene | Braf | 1425693_at | 1.22 ± 0.07 | 1.02 ± 0.09 (6) | LTD | LTP |  |
|  |  | 1435434_at | 0.95 ± 0.03 |  |  |  |  |
|  |  | 1435480_at | 0.92 ± 0.06 |  |  |  |  |
|  |  | 1442749_at | 1.07 ± 0.13 |  |  |  |  |
|  |  | 1445786_at | 1.08 ± 0.22 |  |  |  |  |
|  |  | 1447940_a_at | 1.19 ± 0.18 |  |  |  |  |
|  |  | 1456505_at | 1.11 ± 0.05 |  |  |  |  |
| Calcium channel, voltage-dependent, P/Q type, alpha 1A subunit (Cav2.1) | Cacna1a | 1430408_at | 1.54 ± 0.19 | 0.9 ± 0.24 (6) |  |  | Ca^2+^ |
|  |  | 1450510_a_at | 1.32 ± 0.05* |  |  |  |  |
|  |  | 1459996_at | 1.77 ± 0.05*** |  |  |  |  |
| calbindin-28K | Calb1 | 1417504_at | 1.05 ± 0.03 | 0.56 ± 0.2 (6) |  |  | Ca^2+^ |
|  |  | 1448738_at | 0.98 ± 0.07 |  |  |  |  |
|  |  | 1458836_at | 1.35 ± 0.26 |  |  |  |  |
| calmodulin 1 (CaM1) | Calm1 | 1417365_a_at | 1.06 ± 0.02 | 0.27 ± 0.12* (6) |  | LTP | Ca^2+^ |
|  |  | 1417366_s_at | 1.1 ± 0.04 |  |  |  |  |
|  |  | 1433592_at | 0.97 ± 0.01 |  |  |  |  |
|  |  | 1454611_a_at | 1.03 ± 0.01 |  |  |  |  |
|  |  | 1455571_x_at | 1.01 ± 0.01 |  |  |  |  |
| calmodulin 2 | Calm2 | 1422414_a_at | 1.1 ± 0.03 | 3.21 ± 2.11 (6) |  | LTP | Ca^2+^ |
|  |  | 1423807_a_at | 0.99 ± 0.02 |  |  |  |  |
| calmodulin 3 | Calm3 | 1426710_at | 1 ± 0.03 | 1.36 ± 0.47 (6) |  | LTP | Ca^2+^ |
|  |  | 1438826_x_at | 0.96 ± 0.08 |  |  |  |  |
|  |  | 1450864_at | 0.95 ± 0.06 |  |  |  |  |
| Calcium/calmodulin- dependent protein kinase II, β (CaMK II) | Camk2b | 1448676_at | 1.12 ± 0.02* | 0.74 ± 0.08* (6) |  | LTP | Ca^2+^ |
|  |  | 1455869_at | 1.1 ± 0.07 |  |  |  |  |
| Calcium/calmodulin-dependent protein kinase II, delta | Camk2d | 1422659_at | 0.67 ± 0.04* | 1 ± 0.13 (6) |  | LTP | Ca^2+^ |
|  |  | 1439168_at | 0.6 ± 0.09 |  |  |  |  |
| Calcium/calmodulin- dependent protein kinase II γ (CaMK II) | Camk2g | 1423941_at | 0.76 ± 0.03* | 0.69 ± 0.04*** (6) |  | LTP | Ca^2+^ |
|  |  | 1423942_a_at | 0.83 ± 0.03 |  |  |  |  |
| Cholinergic receptor, muscarinic 4 | Chrm4 |  |  | 0.78 ± 0.14 (6) |  |  |  |
| DEP domain containing 6 | Depdc6 | 1428622_at | 0.9 ± 0.05 | 1.15 ± 0.24 (6) |  |  |  |
|  |  | 1443579_s_at | 0.83 ± 0.04 |  |  |  |  |
|  |  | 1451348_at | 1.23 ± 0.12 |  |  |  |  |
|  |  | 1453571_at | 1.88 ± 0.12** |  |  |  |  |
| dopamine receptor D1A | Drd1a | 1455629_at | 1.06 ± 0.02 | 0.8 ± 0.2 (6) |  |  |  |
|  |  | 1456051_at | 1.22 ± 0.04* |  |  |  |  |
| dopamine receptor 2 (D2R) | Drd2 | 1418950_at | 1.11 ± 0.03 | 0.75 ± 0.13 (6) |  |  |  |
| gamma-aminobutyric acid (GABA-A) receptor, subunit alpha 2 | Gabra2 | 1421738_at | 0.88 ± 0.07 | 0.56 ± 0.17 (9) |  |  |  |
|  |  | 1443865_at | 0.91 ± 0.05 |  |  |  |  |
|  |  | 1446805_at | 1.16 ± 0.22 |  |  |  |  |
|  |  | 1455444_at | 0.94 ± 0.04 |  |  |  |  |
| guanine nucleotide binding protein, alpha inhibiting 1 (Giα1) | Gnai1 | 1427510_at | 0.88 ± 0.03 | 0.54 ± 0.11* (6) | LTD |  |  |
|  |  | 1434440_at | 0.84 ± 0.03* |  |  |  |  |
|  |  | 1454959_s_at | 0.82 ± 0.02* |  |  |  |  |
| guanine nucleotide binding protein, alpha inhibiting 2 (Giα2) | Gnai2 | 1419449_a_at | 0.97 ± 0.05 | 0.56 ± 0.11* (6) | LTD |  |  |
|  |  | 1435652_a_at | 0.93 ± 0.03 |  |  |  |  |
| guanine nucleotide binding protein, alpha inhibiting 3 (Giα3) | Gnai3 | 1428645_at | 0.81 ± 0.03* | 0.45 ± 0.12* (6) | LTD |  |  |
|  |  | 1437225_x_at | 1.09 ± 0.17 |  |  |  |  |
| guanine nucleotide binding protein, alpha o (Goα) | Gnao1 | 1421152_a_at | 1 ± 0.03 | 0.67 ± 0.13 (6) | LTD |  |  |
|  |  | 1448031_at | 1.32 ± 0.06 |  |  |  |  |
| guanine nucleotide binding protein, alpha q polypeptide (Gqα) | Gnaq | 1428938_at | 0.93 ± 0.02 | 0.6 ± 0.12* (6) | LTD | LTP | Ca^2+^ |
|  |  | 1428939_s_at | 0.83 ± 0.03 |  |  |  |  |
|  |  | 1428940_at | 0.86 ± 0.02 |  |  |  |  |
|  |  | 1429559_at | 0.93 ± 0.02 |  |  |  |  |
|  |  | 1446688_at | 1.14 ± 0.14 |  |  |  |  |
|  |  | 1447593_x_at | 0.79 ± 0.03 |  |  |  |  |
|  |  | 1455729_at | 0.85 ± 0.02* |  |  |  |  |
|  |  | 1458159_at | 1.33 ± 0.23 |  |  |  |  |
| GNAS (guanine nucleotide binding protein, alpha stimulating) complex locus (Gsα) | Gnas | 1450186_s_at | 0.88 ± 0.03 | 0.83 ± 0.11 (6) | LTD |  | Ca^2+^ |
| guanine nucleotide binding protein, β 5 (Gβ5) | Gnb5 | 1422208_a_at | 1.06 ± 0.02 | 0.95 ± 0.2 (6) |  |  |  |
|  |  | 1455632_at | 1.17 ± 0.04 |  |  |  |  |
| glutamate receptor, ionotropic, AMPA1 (alpha 1) (GluR1) | Gria1 | 1435239_at | 1.03 ± 0.02 | 0.81 ± 0.11 (6) | LTD | LTP |  |
|  |  | 1448972_at | 1.01 ± 0.02 |  |  |  |  |
|  |  | 1458285_at | 0.92 ± 0.09 |  |  |  |  |
| glutamate receptor, ionotropic, AMPA2 (alpha 2) (GluR2) | Gria2 | 1421970_a_at | 0.89 ± 0.04 | 0.66 ± 0.07** (6) | LTD | LTP |  |
|  |  | 1434146_at | 0.89 ± 0.05 |  |  |  |  |
|  |  | 1453098_at | 1.06 ± 0.1 |  |  |  |  |
| glutamate receptor, ionotropic, AMPA3 (alpha 3) (GluR3) | Gria3 | 1420563_at | 1.03 ± 0.07 | 0.79 ± 0.11 (6) | LTD |  |  |
|  |  | 1434728_at | 0.9 ± 0.05 |  |  |  |  |
| glutamate receptor, ionotropic, NMDA2C (epsilon 3) (NR2C) | Grin2c | 1449245_at | 1.23 ± 0.04* | 0.97 ± 0.2 (6) |  | LTP | Ca^2+^ |
| glutamate receptor, metabotropic 1 (mGlu1) | Grm1 | 1425700_at | 0.9 ± 0.03 | 0.61 ± 0.2 (6) | LTD | LTP | Ca^2+^ |
| Glutamate receptor, metabotropic 5 (mGlu5) | Grm5 | 1443410_at | 1.12 ± 0.19 | 0.82 ± 0.07* (9) | LTD | LTP | Ca^2+^ |
|  |  | 1446679_at | 1.17 ± 0.26 |  |  |  |  |
|  |  | 1455272_at | 0.92 ± 0.04 |  |  |  |  |
|  |  | 1456119_at | 0.92 ± 0.03 |  |  |  |  |
| guanylate cyclase 1, soluble, alpha 3 | Gucy1a3 | 1420533_at | 1.01 ± 0.03 | 0.64 ± 0.13 (8) | LTD |  |  |
|  |  | 1420534_at | 0.92 ± 0.05 |  |  |  |  |
|  |  | 1434141_at | 0.89 ± 0.02* |  |  |  |  |
| inositol 1,4,5- triphosphate receptor 1 (IP3R) | Itpr1 | 1417279_at | 1.14 ± 0.03 | 0.7 ± 0.16 (6) | LTD | LTP | Ca^2+^ |
|  |  | 1457189_at | 1.72 ± 0.27 |  |  |  |  |
|  |  | 1460203_at | 1.07 ± 0.01 |  |  |  |  |
| potassium inwardly- rectifying channel, subfamily J, member 10 | Kcnj10 | 1419601_at | 0.7 ± 0.03* | 0.82 ± 0.16 (6) |  |  |  |
| potassium inwardly- rectifying channel, subfamily J, member 3 | Kcnj3 | 1421468_at | 0.8 ± 0.09 | 0.79 ± 0.23 (6) |  |  |  |
|  |  | 1444336_at | 0.66 ± 0.11 |  |  |  |  |
| Potassium inwardly-rectifying channel, subfamily J, member 6 | Kcnj6 |  |  | 0.6 ± 0.39 (6) |  |  |  |
| potassium inwardly- rectifying channel, subfamily J, member 9 | Kcnj9 | 1450712_at | 0.32 ± 0.01** | 0.81 ± 0.18 (5) |  |  |  |
| v-Ki-ras2 Kirsten rat sarcoma viral oncogene homolog (K-Ras2) | Kras | 1434000_at | 0.87 ± 0.03 | 0.43 ± 0.14* (6) | LTD | LTP |  |
|  |  | 1451979_at | 1.14 ± 0.04 |  |  |  |  |
| mitogen-activated protein kinase kinase 2 | Map2k2 | 1415974_at | 0.88 ± 0.03 | 0.9 ± 0.13 (6) | LTD | LTP |  |
|  |  | 1443436_at | 0.82 ± 0.06 |  |  |  |  |
| mitogen-activated protein kinase 2 | Mapk2 |  |  | 0.94 ± 0.11 (6) |  |  |  |
| mitogen-activated protein kinase 3 (ERK1) | Mapk3 | 1427060_at | 0.83 ± 0.03 | 0.54 ± 0.1** (6) | LTD | LTP |  |
| phosphodiesterase 1A, calmodulin- dependent | Pde1a | 1449298_a_at | 0.49 ± 0.04* | 0.69 ± 0.19 (9) |  |  | Ca^2+^ |
| phosphodiesterase 4B, cAMP specific (PDE4b) | Pde4b | 1422473_at | 1.02 ± 0.03 | 0.66 ± 0.12* (6) |  |  |  |
|  |  | 1422474_at | 0.99 ± 0.02 |  |  |  |  |
|  |  | 1442700_at | 1.22 ± 0.16 |  |  |  |  |
|  |  | 1447237_at | 1.61 ± 0.71 |  |  |  |  |
| phosphodiesterase 7B | Pde7b | 1421353_at | 1.41 ± 0.06* | 0.74 ± 0.21 (6) |  |  |  |
|  |  | 1450213_at | 1.27 ± 0.04* |  |  |  |  |
| phosphodiesterase 8B | Pde8b | 1435741_at | 1.26 ± 0.02*** | 0.77 ± 0.12 (6) |  |  |  |
|  |  | 1437989_at | 1.14 ± 0.03 |  |  |  |  |
| phospholipase A2 receptor 1 | Pla2r1 |  |  | 0.83 ± 0.21 (6) |  |  |  |
| phospholipase C, beta 1 (Plcβ1) | Plcb1 | 1421170_a_at | 1.12 ± 0.03 | 0.53 ± 0.11* (6) | LTD | LTP | Ca^2+^ |
|  |  | 1425600_a_at | 0.86 ± 0.05 |  |  |  |  |
|  |  | 1425781_a_at | 1.1 ± 0.07 |  |  |  |  |
|  |  | 1425782_at | 0.97 ± 0.15 |  |  |  |  |
|  |  | 1435043_at | 0.79 ± 0.02* |  |  |  |  |
| protein phosphatase 1, regulatory (inhibitor) subunit 12A (PP1) | Ppp1r12a | 1429487_at | 0.87 ± 0.01** | 0.97 ± 0.09 (6) |  | LTP |  |
|  |  | 1437734_at | 0.96 ± 0.04 |  |  |  |  |
|  |  | 1437735_at | 0.98 ± 0.03 |  |  |  |  |
|  |  | 1444762_at | 1.16 ± 0.15 |  |  |  |  |
|  |  | 1453163_at | 1.43 ± 0.06* |  |  |  |  |
| protein phosphatase 1, regulatory (inhibitor) subunit 1B (DARPP32) | Ppp1r1b | 1451331_at | 1.09 ± 0.03 | 0.76 ± 0.05** (6) |  |  |  |
| protein phosphatase 2 (formerly 2A), regulatory subunit A (PR 65), alpha isoform (PP2A) | Ppp2r1a | 1415819_a_at | 0.97 ± 0.03 | 0.87 ± 0.07 (6) | LTD |  |  |
|  |  | 1438174_x_at | 1 ± 0.03 |  |  |  |  |
|  |  | 1438383_x_at | 0.95 ± 0.01 |  |  |  |  |
|  |  | 1438991_x_at | 0.94 ± 0.01 |  |  |  |  |
|  |  | 1455929_x_at | 0.94 ± 0.01* |  |  |  |  |
| protein phosphatase 3, catalytic subunit, beta isoform (PP2B) | Ppp3cb | 1427468_at | 0.96 ± 0.02 | 0.97 ± 0.11 (6) |  | LTP | Ca^2+^ |
|  |  | 1428473_at | 0.97 ± 0.01 |  |  |  |  |
|  |  | 1428474_at | 0.77 ± 0.03* |  |  |  |  |
|  |  | 1433835_at | 0.89 ± 0.03 |  |  |  |  |
|  |  | 1446149_at | 1.14 ± 0.16 |  |  |  |  |
|  |  | 1459378_at | 1.04 ± 0.18 |  |  |  |  |
| protein phosphatase 3, regulatory subunit B, alpha isoform (calcineurin B, type I) (PP2B) | Ppp3r1 | 1421786_at | 1.32 ± 0.13 | 0.97 ± 0.08 (6) |  | LTP | Ca^2+^ |
|  |  | 1433591_at | 0.98 ± 0.02 |  |  |  |  |
|  |  | 1450368_a_at | 1.27 ± 0.09 |  |  |  |  |
| protein phosphatase 3, regulatory subunit B, alpha isoform (calcineurin B, type II) (PP2B) | Ppp3r2 |  |  | 1.01 ± 0.4 (6) |  | LTP | Ca^2+^ |
| protein kinase, cAMP dependent, catalytic, alpha (PKA) | Prkaca | 1447720_x_at | 0.95 ± 0.03 | 0.9 ± 0.17 (6) |  | LTP | Ca^2+^ |
|  |  | 1450519_a_at | 0.85 ± 0.04 |  |  |  |  |
| protein kinase, cAMP dependent, catalytic, beta (PKA) | Prkacb | 1420610_at | 1.18 ± 0.07 | 0.67 ± 0.11* (9) |  | LTP | Ca^2+^ |
|  |  | 1420611_at | 1.02 ± 0.01 |  |  |  |  |
| protein kinase C, beta 1 (PKC) | Prkcb1 | 1423478_at | 1.02 ± 0.01 | 0.51 ± 0.09** (6) | LTD | LTP | Ca^2+^ |
|  |  | 1438981_at | 1.48 ± 0.22 |  |  |  |  |
|  |  | 1443144_at | 1.11 ± 0.23 |  |  |  |  |
|  |  | 1459674_at | 1.69 ± 0.23 |  |  |  |  |
|  |  | 1460419_a_at | 0.91 ± 0.01* |  |  |  |  |
| protein kinase C, iota | Prkci | 1417410_s_at | 1.48 ± 0.04*** | 0.94 ± 0.29 (6) |  |  |  |
| protein kinase, cGMP-dependent, type I | Prkg1 | 1444232_at | 0.79 ± 0.05 | 1.13 ± 0.37 (6) | LTD |  |  |
|  |  | 1445807_at | 0.48 ± 0.14 |  |  |  |  |
| RAS related protein 1b | Rap1b | 1435518_at | 1.23 ± 0.04* | 0.97 ± 0.04 (6) |  | LTP |  |
|  |  | 1435519_at | 1.06 ± 0.02 |  |  |  |  |
|  |  | 1455349_at | 0.98 ± 0.03 |  |  |  |  |
| Rap guanine nucleotide exchange factor (GEF) 3 | Rapgef3 | 1424470_a_at | 0.79 ± 0.03 | 0.66 ± 0.1* (9) |  | LTP |  |
|  |  | 1437012_x_at | 1.02 ± 0.04 |  |  |  |  |
| regulator of G- protein signaling 4 | Rgs4 | 1416286_at | 1.24 ± 0.03** | 0.99 ± 0.22 (6) |  |  |  |
|  |  | 1416287_at | 1.65 ± 0.1** |  |  |  |  |
|  |  | 1448285_at | 1.71 ± 0.13* |  |  |  |  |
| regulator of G- protein signaling 9 (RGS9) | Rgs9 | 1418691_at | 1.3 ± 0.04** | 1.47 ± 0.2* (6) |  |  |  |
|  |  | 1439635_at | 3.18 ± 0.73* |  |  |  |  |
| Regulator of G- protein signaling 9 binding protein (R7BP) | Rgs9bp |  |  | 0.59 ± 0.23 (6) |  |  |  |
| ryanodine receptor 1, skeletal muscle | Ryr1 | 1427306_at | 0.46 ± 0.04* | 0.19 ± 0.04*** (6) | LTD |  | Ca^2+^ |
|  |  | 1457347_at | 0.54 ± 0.06* |  |  |  |  |
| ryanodine receptor 2, cardiac | Ryr2 | 1421126_at | 1.08 ± 0.15 | 0.71 ± 0.22 (6) |  |  | Ca^2+^ |
|  |  | 1445498_at | 1.21 ± 0.18 |  |  |  |  |
|  |  | 1450123_at | 1.02 ± 0.04 |  |  |  |  |
| ryanodine receptor 3 | Ryr3 | 1427427_at | 1.18 ± 0.05 | 0.4 ± 0.2 (6) |  |  | Ca^2+^ |
|  |  | 1452533_at | 1.32 ± 0.1 |  |  |  |  |

**Table S5.**

| **transcript** | **gene symbol** | **Probe Set ID** | **microaray** |
| --- | --- | --- | --- |
| ATP-binding cassette, sub-family C (CFTR/MRP), member 5 | Abcc5 | 1438056_x_at | 1.53 ± 0.06** |
| Adenosine deaminase, RNA-specific, B2 | Adarb2 | 1446233_at | 2.1 ± 0.16* |
| Adenosine deaminase, RNA-specific, B2 | Adarb2 | 1458670_at | 1.66 ± 0.05*** |
| AF4/FMR2 family, member 1 | Aff1 | 1418135_at | 1.59 ± 0.11* |
| ankyrin repeat and KH domain containing 1 | Ankhd1 | 1455759_a_at | 1.55 ± 0.13* |
| aquaporin 7 | Aqp7 | 1418849_x_at | 0.15 ± 0.04*** |
| activity regulated cytoskeletal-associated protein | Arc | 1418687_at | 1.88 ± 0.2* |
| aryl hydrocarbon receptor nuclear translocator 2 | Arnt2 | 1420669_at | 1.55 ± 0.12* |
| aryl hydrocarbon receptor nuclear translocator-like 2 | Arntl2 | 1429688_at | 0.45 ± 0.05** |
| activating transcription factor 6 | Atf6 | 1456021_at | 1.63 ± 0.11** |
| ATPase, Na+/K+ transporting, alpha 2 polypeptide | Atp1a2 | 1434893_at | 1.84 ± 0.14** |
| ATPase, class I, type 8B, member 1 | Atp8b1 | 1455396_at | 1.65 ± 0.04*** |
| expressed sequence AU015892 | AU015892 | 1446830_at | 1.62 ± 0.09** |
| RIKEN cDNA B230312I18 gene | B230312I18Rik | 1437212_at | 0.41 ± 0.02*** |
| UDP-Gal:betaGal beta 1,3-galactosyltransferase, polypeptide 6 | B3galt6 | 1435252_at | 3.64 ± 2.33** |
| beta-1,4-N-acetyl-galactosaminyl transferase 1 | B4galnt1 | 1425363_at | 1.95 ± 0.33* |
| Bcl-associated death promoter | Bad | 1416583_at | 1.63 ± 0.16* |
| cDNA sequence BC051142 | BC051142 | 1437264_at | 1.74 ± 0.19** |
| RIKEN cDNA C030013G03 gene | C030013G03Rik | 1442754_at | 1.83 ± 0.12** |
| C1q domain containing 2 | C1qdc2 | 1439422_a_at | 2.3 ± 0.14*** |
| Calcium channel, voltage-dependent, P/Q type, alpha 1A subunit | Cacna1a | 1459996_at | 1.77 ± 0.05*** |
| carbonic anyhydrase 12 | Car12 | 1428485_at | 1.57 ± 0.06*** |
| CDK5 regulatory subunit associated protein 1-like 1 | Cdkal1 | 1459314_at | 3.11 ± 0.47** |
| centrosomal protein 152 | Cep152 | 1427496_at | 1.51 ± 0.15* |
| CDC28 protein kinase regulatory subunit 2 | Cks2 | 1417457_at | 1.55 ± 0.11* |
| chloride channel calcium activated 3 | Clca3 | 1459889_at | 0.36 ± 0.06* |
| cryptochrome 1 (photolyase-like) | Cry1 | 1433733_a_at | 1.56 ± 0.11* |
| cytochrome P450, family 2, subfamily a, polypeptide 4 /// cytochrome P450, family 2, subfamily a, polypeptide 5 | Cyp2a4 /// Cyp2a5 | 1422230_s_at | 1.93 ± 0.11** |
| Death associated protein kinase 1 | Dapk1 | 1445032_at | 1.94 ± 0.23* |
| Duffy blood group, chemokine receptor | Darc | 1432273_a_at | 1.54 ± 0.05*** |
| DEP domain containing 6 | Depdc6 | 1453571_at | 1.88 ± 0.12** |
| deoxyguanosine kinase | Dguok | 1425228_a_at | 1.55 ± 0.12* |
| deiodinase, iodothyronine, type II | Dio2 | 1418938_at | 1.52 ± 0.09* |
| DnaJ (Hsp40) homolog, subfamily A, member 4 | Dnaja4 | 1418592_at | 1.51 ± 0.06** |
| Dystonin | Dst | 1442395_at | 1.52 ± 0.12* |
| dual specificity phosphatase 1 | Dusp1 | 1448830_at | 1.51 ± 0.07* |
| dual specificity phosphatase 11 (RNA/RNP complex 1-interacting) | Dusp11 | 1452594_at | 1.73 ± 0.11* |
| dual specificity phosphatase 14 | Dusp14 | 1431422_a_at | 1.7 ± 0.08** |
| dual specificity phosphatase 3 (vaccinia virus phosphatase VH1-related) | Dusp3 | 1425608_at | 1.57 ± 0.06*** |
| ER degradation enhancer, mannosidase alpha-like 2 | Edem2 | 1423695_at | 1.52 ± 0.11* |
| eukaryotic translation initiation factor 2C, 4 | Eif2c4 | 1429779_at | 1.54 ± 0.06** |
| empty spiracles homolog 2 (Drosophila) opposite strand | Emx2os | 1456559_at | 1.59 ± 0.2* |
| ectonucleoside triphosphate diphosphohydrolase 5 | Entpd5 | 1451765_a_at | 1.5 ± 0.12* |
| extra spindle poles-like 1 (S. cerevisiae) | Espl1 | 1433862_at | 1.5 ± 0.08* |
| Exosome component 4 | Exosc4 | 1441004_at | 1.8 ± 0.19* |
| Fibroblast growth factor 3 | Fgf3 | 1441914_x_at | 2.22 ± 0.25* |
| FGFR1 oncogene partner 2 | Fgfr1op2 | 1431020_a_at | 2.21 ± 0.13* |
| FGFR1 oncogene partner 2 | Fgfr1op2 | 1441460_at | 1.68 ± 0.13* |
| FBJ osteosarcoma oncogene | Fos | 1423100_at | 1.76 ± 0.15* |
| fos-like antigen 2 /// similar to fos-like antigen 2 | Fosl2 /// LOC634417 | 1437247_at | 1.52 ± 0.09* |
| growth arrest and DNA-damage-inducible, gamma interacting protein 1 | Gadd45gip1 | 1417619_at | 0.46 ± 0.03** |
| UDP-N-acetyl-alpha-D-galactosamine: polypeptide N-acetylgalactosaminyltransferase 7 | Galnt7 | 1425581_s_at | 1.64 ± 0.12* |
| glucosaminyl (N-acetyl) transferase 1, core 2 | Gcnt1 | 1460431_at | 1.57 ± 0.14* |
| glucosaminyl (N-acetyl) transferase 2, I-branching enzyme | Gcnt2 | 1451733_at | 1.61 ± 0.12* |
| glial cell line derived neurotrophic factor family receptor alpha 2 | Gfra2 | 1433716_x_at | 0.48 ± 0.07* |
| gene model 996, (NCBI) | Gm996 | 1436445_at | 0.44 ± 0.05** |
| glia maturation factor, gamma | Gmfg | 1419194_s_at | 1.73 ± 0.29* |
| guanine nucleotide binding protein, beta 4 | Gnb4 | 1419469_at | 0.33 ± 0.02** |
| glutamate oxaloacetate transaminase 2, mitochondrial /// similar to Aspartate aminotransferase, mitochondrial precursor (Transaminase A) (Glutamate oxaloacetate transaminase 2) | Got2 /// LOC640847 | 1447768_at | 0.44 ± 0.06** |
| glypican 6 | Gpc6 | 1437417_s_at | 1.71 ± 0.09*** |
| G protein-coupled receptor 178 | Gpr178 | 1428117_x_at | 1.68 ± 0.26* |
| gene regulated by estrogen in breast cancer protein | Greb1 | 1439568_at | 1.57 ± 0.15* |
| homeodomain interacting protein kinase 3 | Hipk3 | 1419191_at | 1.76 ± 0.42* |
| heat shock protein 8 /// similar to heat shock protein 8 | Hspa8 | 1431182_at | 1.68 ± 0.13* |
| insulin-like growth factor binding protein 6 | Igfbp6 | 1417933_at | 1.98 ± 0.09** |
| interleukin-1 receptor-associated kinase 3 | Irak3 | 1435040_at | 2.75 ± 0.89* |
| interferon regulatory factor 6 | Irf6 | 1418301_at | 0.44 ± 0.06** |
| potassium inwardly-rectifying channel, subfamily J, member 9 | Kcnj9 | 1450712_at | 0.32 ± 0.01** |
| kinesin family member 6 | Kif6 | 1442238_a_at | 1.56 ± 0.13* |
| kelch domain containing 5 | Klhdc5 | 1426988_at | 2.14 ± 0.09*** |
| kringle containing transmembrane protein 1 | Kremen1 | 1445252_at | 1.76 ± 0.17* |
| leucyl-tRNA synthetase, mitochondrial | Lars2 | 1435682_at | 2.12 ± 0.26* |
| Similar to reduced expression 2 | LOC195531 | 1436386_x_at | 2.85 ± 0.62** |
| hypothetical protein LOC667085 /// hypothetical protein LOC672049 | LOC667085 /// LOC672049 | 1442234_at | 0.42 ± 0.04** |
| mitogen activated protein kinase kinase kinase 12 | Map3k12 | 1438908_at | 1.58 ± 0.07* |
| nucleolar protein 3 (apoptosis repressor with CARD domain) | Nol3 | 1444786_at | 0.5 ± 0.07* |
| nephroblastoma overexpressed gene | Nov | 1426852_x_at | 0.31 ± 0.04* |
| neuronal PAS domain protein 4 | Npas4 | 1459372_at | 1.92 ± 0.25* |
| N-acetylneuraminate pyruvate lyase | Npl | 1424265_at | 1.57 ± 0.03*** |
| neuronal pentraxin 2 | Nptx2 | 1420720_at | 1.77 ± 0.07*** |
| oxysterol binding protein-like 3 | Osbpl3 | 1428484_at | 1.62 ± 0.09** |
| proliferation-associated 2G4 | Pa2g4 | 1450854_at | 1.58 ± 0.06** |
| phosphodiesterase 1A, calmodulin-dependent | Pde1a | 1449298_a_at | 0.49 ± 0.04* |
| pam, highwire, rpm 1 | Phr1 | 1458870_x_at | 1.53 ± 0.08* |
| proviral integration site 1 | Pim1 | 1435458_at | 2.42 ± 0.62** |
| pleckstrin homology domain containing, family G (with RhoGef domain) member 4 | Plekhg4 | 1457145_at | 1.72 ± 0.07** |
| POU domain, class 3, transcription factor 2 | Pou3f2 | 1457734_at | 1.52 ± 0.1* |
| peroxisome proliferator activated receptor binding protein | Pparbp | 1439408_a_at | 1.74 ± 0.21* |
| protein phosphatase 1, regulatory (inhibitor) subunit 14A | Ppp1r14a | 1418086_at | 0.38 ± 0.02*** |
| peroxiredoxin 2 | Prdx2 | 1430979_a_at | 0.19 ± 0.01*** |
| proline-rich transmembrane protein 3 | Prrt3 | 1443919_at | 1.63 ± 0.09** |
| RAS-related C3 botulinum substrate 1 | Rac1 | 1437674_at | 0.47 ± 0.04** |
| retinol binding protein 4, plasma | Rbp4 | 1426225_at | 1.67 ± 0.13* |
| regulator of G-protein signaling 4 | Rgs4 | 1416287_at | 1.65 ± 0.1** |
| regulator of G-protein signaling 4 | Rgs4 | 1448285_at | 1.71 ± 0.13* |
| regulator of G-protein signaling 9 | Rgs9 | 1439635_at | 3.18 ± 0.73* |
| Rho family GTPase 3 | Rnd3 | 1416701_at | 1.66 ± 0.18* |
| ribosomal protein S24 | Rps24 | 1455195_at | 1.75 ± 0.08* |
| Restin (Reed-Steinberg cell-expressed intermediate filament-associated protein) | Rsn | 1444750_at | 1.56 ± 0.08* |
| ryanodine receptor 1, skeletal muscle | Ryr1 | 1427306_at | 0.46 ± 0.04* |
| solute carrier family 25, member 27 | Slc25a27 | 1458119_at | 1.94 ± 0.18* |
| SET and MYND domain containing 5 | Smyd5 | 1447749_at | 1.51 ± 0.07** |
| syntrophin, gamma 2 | Sntg2 | 1418789_at | 1.77 ± 0.17* |
| spectrin beta 2 | Spnb2 | 1451830_a_at | 1.51 ± 0.12* |
| Sprouty-related, EVH1 domain containing 2 | Spred2 | 1441415_at | 1.75 ± 0.12* |
| TAO kinase 2 | Taok2 | 1438208_at | 1.53 ± 0.07* |
| transforming growth factor, beta receptor I | Tgfbr1 | 1420894_at | 1.73 ± 0.11** |
| transducin-like enhancer of split 6, homolog of Drosophila E(spl) | Tle6 | 1448727_at | 1.94 ± 0.13** |
| tropomyosin 1, alpha | Tpm1 | 1456623_at | 1.95 ± 0.25* |
| tubulin tyrosine ligase like family 9 | Ttll9 | 1431029_at | 0.49 ± 0.04* |
| transthyretin | Ttr | 1454608_x_at | 0.14 ± 0.03* |
| transthyretin | Ttr | 1455913_x_at | 0.21 ± 0.03* |
| transthyretin | Ttr | 1459737_s_at | 0.23 ± 0.03* |
| unc-5 homolog D (C. elegans) | Unc5d | 1440484_at | 0.42 ± 0.03*** |
| UTP18, small subunit (SSU) processome component, homolog (yeast) | Utp18 | 1447266_at | 2.37 ± 1.63* |
| WD repeats and SOF domain containing 1 | Wdsof1 | 1458778_at | 2.11 ± 0.23* |
| EST X83328 | X83328 | 1417241_at | 1.53 ± 0.04** |
| Yip1 domain family, member 4 | Yipf4 | 1426417_at | 0.46 ± 0.03*** |
| zinc finger protein 14 | Zfp14 | 1441619_at | 1.87 ± 0.1*** |
